# High-Fidelity Coding with Correlated Neurons

**DOI:** 10.1371/journal.pcbi.1003970

**Published:** 2014-11-20

**Authors:** Rava Azeredo da Silveira, Michael J. Berry

**Affiliations:** 1 Department of Physics, Ecole Normale Supérieure, Paris, France; 2 Laboratoire de Physique Statistique, Centre National de la Recherche Scientifique, Université Pierre et Marie Curie, Université Denis Diderot, Paris, France; 3 Princeton Neuroscience Institute, Princeton University, Princeton, New Jersey, United States of America; 4 Department of Molecular Biology, Princeton University, Princeton, New Jersey, United States of America; Université Paris Descartes, Centre National de la Recherche Scientifique, France

## Abstract

Positive correlations in the activity of neurons are widely observed in the brain. Previous studies have shown these correlations to be detrimental to the fidelity of population codes, or at best marginally favorable compared to independent codes. Here, we show that positive correlations can enhance coding performance by astronomical factors. Specifically, the probability of discrimination error can be suppressed by many orders of magnitude. Likewise, the number of stimuli encoded—the capacity—can be enhanced more than tenfold. These effects do not necessitate unrealistic correlation values, and can occur for populations with a few tens of neurons. We further show that both effects benefit from heterogeneity commonly seen in population activity. Error suppression and capacity enhancement rest upon a pattern of correlation. Tuning of one or several effective parameters can yield a limit of perfect coding: the corresponding pattern of positive correlation leads to a ‘lock-in’ of response probabilities that eliminates variability in the subspace relevant for stimulus discrimination. We discuss the nature of this pattern and we suggest experimental tests to identify it.

## Author Summary

Traditionally, sensory neuroscience has focused on correlating inputs from the physical world with the response of a single neuron. Two stimuli can be distinguished solely from the response of one neuron if one stimulus elicits a response and the other does not. But as soon as one departs from extremely simple stimuli, single-cell coding becomes less effective, because cells often respond weakly and unreliably. High fidelity coding then relies upon populations of cells, and correlation among those cells can greatly affect the neural code. While previous theoretical studies have demonstrated a potential coding advantage of correlation, they allowed only a marginal improvement in coding power. Here, we present a scenario in which a pattern of correlation among neurons in a population yields an improvement in coding performance by several orders of magnitude. By “improvement” we mean that a neural population is much better at both distinguishing stimuli and at encoding a large number of them. The scenario we propose does not invoke unrealistic values of correlation. What is more, it is even effective for small neural populations and in subtle cases in which single-cell coding fails utterly. These results demonstrate a previously unappreciated potential for correlated population coding.

## Introduction

Many of the classic studies relating behavior to the activity of neurons, such as studies of single photon counting, have focused on behaviors that are near the threshold of perception [1], [2], [3], [4], [5], where performance is uncertain and can suffer a substantial error rate. One of the surprises of these studies is that in this limit, the variability of single neurons often matches the variability in performance, such that single neurons can account for the behavior [4], [6], [7]. However, most of our everyday visual experience involves judgments made with great accuracy and certainty. As is illustrated by phrases like “seeing is believing” and Shakespeare's “ocular proof,” we often dismiss any doubt about an aspect of the world once it is perceived visually. In this ‘high-fidelity’ limit, perception must cope with single neuron variability by relying upon populations of neurons. Our visual system not only yields perception with certainty, but it also allows us to make complex judgments very rapidly—a fact that places additional constraints on the population neural code [8], [9].

In a neural population, correlations in the activity of neurons provide additional variables with which information can be represented. While details may vary from one neural circuit to another, a fairly common pattern of correlation is observed across many brain regions, including the retina, LGN, cerebral cortex, and cerebellum [10], [11], [12], [13], [14], [15], [16], [17]. Correlations vary from pair to pair, with a positive mean and a standard deviation comparable to the mean [18], [19], [20], [21], [22], [23] (but see Ref. [24]). Whereas noise correlations adopt moderate values in the retina and may not contribute much to the coding accuracy, their larger values—possibly reflecting the underlying recurrent neural dynamics—in cortex suggest that, there, they may have greater incidence upon coding properties.

How do these affect coding? This question has been investigated by a number of authors [25], [26], [27], [28], [29], [30], [31], [32], [33], [34], [35], [36], [37], [38], [39], [40], who find that in many cases positive correlations are detrimental to coding performance; in some cases, however, positive correlations can enhance the coding performance of a neural population. Using specific choices of neural response and correlation properties, this effect was probed quantitatively in models of pairs of neurons, small populations, or large populations. In all these cases, the presence of positive correlation boosted coding performance to a relatively modest degree: the mutual (Shannon) information or the Fisher information (depending on the study) in the correlated population exceeded that in the equivalent independent population by a factor of 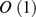. For typical choices of correlation values, the improvement was calculated to be 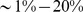. These results can be translated into the units of capacity used in this study and correspond to an improvement of a fraction of a percent to a few percents (see Discussion below), which in turn correspond to a negligible increase of the information encoded per neuron. Recently [41], [42] (see also Ref. [31], [37]), the Fisher information and related quantities were revisited for more general cases of either the tuning properties of neurons [41] or the structure of pairwise correlation [42]. In the resulting picture, earlier statements about the detrimental effect of positive correlation are nuanced. These analyses demonstrate, in particular, that correlation can be helpful in the presence of neuron-to-neuron variability of the tuning curve [37], [41] or when correlation adopts more complicated structures than the ones considered in earlier work [42].

Here, we focus upon the case of stimulus-independent correlation. We pose the problem in much the same way as it was posed in a number of earlier studies [30], [31], [32], [33], [36], [37], [38], [39], [40] extending the work of Abbott and Dayan [28], and which itself can be traced, possibly, to similar ideas that appeared earlier in the literature (see, e.g., [25], [26]). Namely, we ask how the structure of the correlation – specifically, of the covariance matrix – affects coding performance. We exploit the same idea that was used in the papers just referenced: correlation can enhance coding performance by a simple mechanism—relegating the variability of neural response into non-informative modes of the population activity. For a more precise statement, note that, because of variability each stimulus is represented by a distribution of response patterns in the population, and the overlap between neighboring distributions results in coding ambiguity. While, generically, positive correlations broaden response distributions, depending upon the interplay between the mean response properties of neurons and their correlations, probability distributions can be, instead, deformed by correlations in such a way as to suppress overlap.

While much of the earlier literature on this topic is set in the context of continuous stimuli, here we focus upon the case of discrete stimuli (‘categorical perception’). (This does not represent a fundamental conceptual shift, but the case of discrete stimuli begs for different mathematical objects, such as the discrimination error rate and the information capacity, rather than information theoretic quantities which depend upon a continuous stimulus such as the Fisher information.) First, we shall investigate the coding performance in a discrimination task that involves two stimuli. In this case, by construction, a subset of the neural population will respond preferentially to one stimulus, while the remaining neurons in the population will be more responsive to the other stimulus; hence, this ‘staggered preference’ is assumed without any loss of generality. (This ‘staggered preference’, also, plays a similar role to that of putative negative signal correlations in earlier work – see our comments on this issue, below.) In this context, we shall demonstrate that some patterns of positive correlations can serve to suppress discrimination errors by many orders of magnitude. Second, we shall consider the more general case in which the discrimination task involves a large number of stimuli—the question then becomes one of capacity: how many stimuli can be encoded by a population of neurons at very low error rates? We shall show that the capacity can be enhanced significantly by the presence of correlation; specifically, the information per neuron can be boosted by factors of 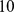 (or even greater), as compared with an equivalent independent population. Interestingly, an astronomical enhancement in coding performance does not require a large population; it can occurs in small populations with tens or hundreds of neurons and, also, it can occur in cases in which independent coding breaks down entirely. Along the way, we shall discuss some auxiliary results, such as the favorable role of neuron-to-neuron variability in the response properties and a possible experimental approach to our ideas, as well as quantitative relations between our work and earlier results.

If one or several parameters are fine-tuned, the system reaches a ‘lock-in’ limit in which coding can become perfect: the distribution of population responses becomes ‘compressed’ into a lower-dimensional object. While in this limit some population patterns are forbidden, population responses are still variable and pairwise correlation coefficients can have moderate values similar to the ones measured experimentally. If the population is close to this singular, fine-tuned limit, then even though coding is not perfect one can obtain an astronomical enhancement of the coding performance as compared to that of a population of independent neurons. Furthermore, this enhancement is robust to variations in the additional (‘untuned’) parameters in the system. The resulting picture results from a collective phenomenon. Earlier work exploited the basic mechanism in models in which the role of correlation involved, in effect, pairs or very small numbers of neurons. In our work, we invoke a pooling mechanism: even in the presence of only weak correlations, a moderately small sub-population can behave like a nearly deterministic, ‘macro-neuron’. Thus, at the cost of losing some amount of coding performance by having homogeneous pools within the population, we obtain a tremendous enhancement because the variability in the informative directions can be severely suppressed.

## Results

Our results amount to the answers to two complementary questions. Given a pair sensory stimuli, how well can a population of correlated neurons discriminate between them? Or, more precisely, what is the discrimination error rate? Conversely, given a discrimination error rate, what is the capacity of a correlated population? That is, how many stimuli can it encode with tolerable error? In natural situations, discrimination errors are exceedingly rare and, hence, neural populations are expected to achieve very low error rates. (See Discussion for a detailed argument and quantitative estimates of low error rates.) The present work is set in this low-error regime.

Since we are interested in rapid coding, we focus on short time windows. The biophysical time scale of neurons—a few tens of milliseconds—affords us with a natural choice. This time scale also happens to correspond to the spike timing jitter of individual neurons in the early visual pathway in response to a natural movie clip [43]. We consider short time bins in which each neuron can only fire one spike or none at all. (This last assumption is not essential; in the more general case in which many spikes can fit in a time bin, our qualitative conclusions remain unchanged or may even become stronger. Furthermore, in some examples we shall assume a relatively high firing rate—say, 50%. In those cases we can still assume a binary output by identifying all cases in which there is at least one spike per time bin, i.e., by saying that a cell is either silent or firing in a time bin. A perceptron-like decoder can implement this identification by an appropriate saturating non-linearity which collapses unto the same output all inputs with one or more spikes.) The situation we have in mind is one in which a stimulus is presented once every time bin, and the corresponding population response is recorded.

### Positive correlations can suppress discrimination error rates by orders of magnitude

**Figure 1:**
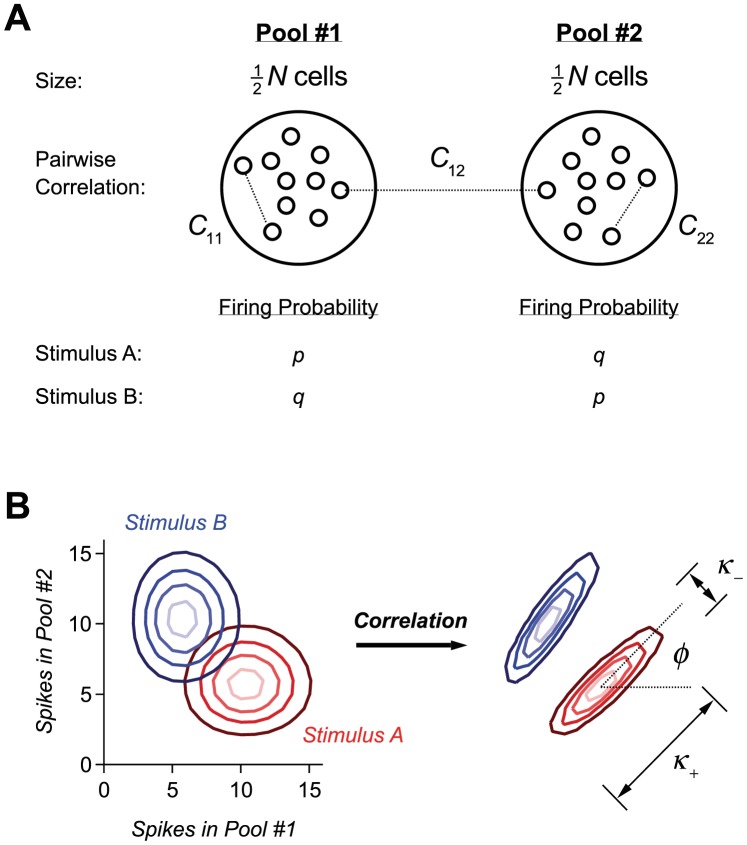
Simple model of a population code. **A.** Schematics of our model with two pools with 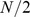 neurons each. Correlation within Pool 1 is 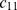 for all pairs; correlation within Pool 2 is 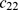 for all pairs; correlation between the two pools is 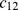 for all pairs. Firing probability in a single window of time for Pool 1 is 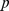 for Target and 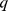 for Distracter; firing probabilities are the opposite for Pool 2. **B.** Probability contours (lightest shade represents highest probability) for Target stimulus (red) and Distracter (blue) stimuli in the case of independent neurons (left). Correlation can shrink the distribution along the line separating them and extend the distribution perpendicular to their separation (right). Variances along the two principle axes are denoted by 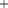 and 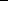; the angle between the long axis and the horizontal line is denoted by 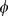. Variances along the axes of Pool 1 and 2 are denoted by 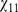 and 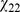, respectively; the variance across Pools 1 and 2 is denoted by 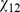.

We consider two stimuli, which we henceforth refer to as Target and Distracter, and we consider a situation in which these have to be discriminated by the response of a neural population in a short time window during which each neuron fires 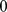 or 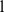 spike. Each neuron is bound to respond more vigorously on average either to Target or to Distracter. Thus, it is natural to divide the 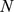-neuron population into two pools of neurons (“Pool 1” and “Pool 2”), each more responsive to one of the two stimuli, as it has been done customarily in studies on stimulus discrimination (see, e.g., [4]). For the sake of simplicity, in this 2-Pool model we allocate 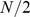 neurons to each pool (Fig. 1A). We denote by 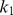 and 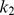 the number of active neurons in Pools 1 and 2 respectively. We start with a symmetric case: neurons in Pools 1 and 2 respond with firing rates 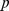 and 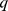 respectively to the Target and, conversely, with firing rates 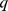 and 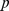 respectively to the Distracter. Moreover, correlations in the activity of pairs of neurons may take different values within Pool 1 (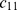), within Pool 2 (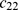), and across pools (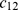). We denote by 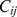 the elements of the covariance matrix and by 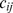 the normalized pairwise correlations; normalized values are often quoted in the literature and present the advantage of being bounded by 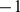 and 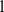. (See Materials and Methods for mathematical definitions.) While we shall present most of our quantitative results for symmetric choices of the parameters, our qualitative conclusions hold in general.

In the discrimination case just outlined, between two individual stimuli (e.g., a given black cat and a given tabby cat), any correlations in question are what is often referred to in the literature as noise correlations; these reflect the dynamics of the neural network, not any structure inherent to stimuli. In order to relate this setup to that of earlier studies involving continuous stimuli, we mention that, although we cannot define signal correlation, here, the ‘staggered preference’ in the population (i.e., the fact that different pools of neurons respond preferentially to different stimuli) plays a similar role to that of negative signal correlation in earlier work.

One can also define a discrimination task between an individual stimulus and a stimulus category (e.g., a given black and all other cats) or between two stimulus categories (e.g., all black cats and all tabby cats). In the case of these problems, the correlations at play are combinations of noise correlations and signal correlations; the latter reflect both the response properties of neurons and the structure of the stimulus ensemble. At the level of the mathematical treatments in our study, the distinction between noise and signal correlations is irrelevant: our derivations make use of the matrix of activity covariances without reference to their origin. The same goes for the actual problem faced by the brain: a readout neuron does not ‘know’ whether the correlations it sees are noise or signal correlations. However, for the sake of conceptual clarity, we shall phrase our discrimination problem as one between two individual stimuli; thus, the reader can think of the elements of the covariance matrix and the normalized correlation coefficients as representing noise correlations.

If 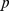 is larger than 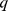, Pool 1 consists of the neurons ‘tuned’ to Target while Pool 2 consists of the neurons ‘tuned’ to Distracter. A useful visual representation of the probability distributions of responses to Target and Distracter makes use of contour lines (Fig. 1B). In the case of independent neurons (with 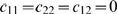), the principal axes of the two distributions are horizontal and vertical, and their contour lines are nearly circular unless 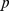 or 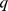 take extreme values. As a result, the overlap between the two distributions tends to be significant (Fig. 1B), with the consequence of a non-negligible coding error rate. In such a situation, positive correlations can improve coding by causing the distributions to elongate along the diagonal and, conversely, to shrink along the line that connects the two centers (Fig 1B).

**Figure 2:**
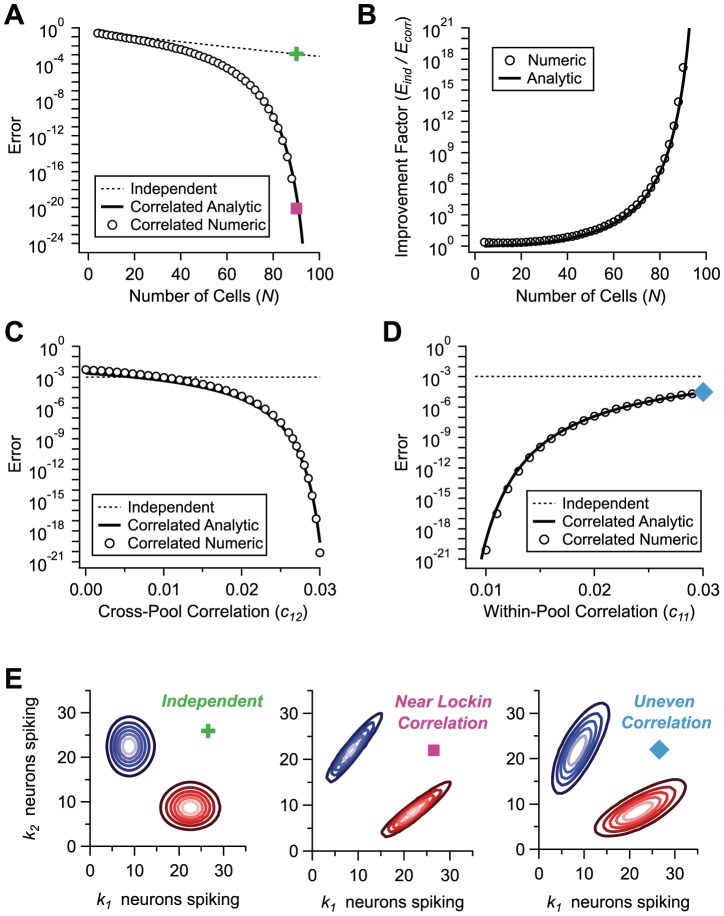
Positive correlation can dramatically suppress the error. **A.** Probability of discrimination error for a 2-Pool model of a neural population, as a function of the number of neurons, 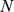, for independent (dashed; all 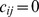) and correlated (circles) populations; parameters are 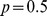, 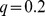 for both, and 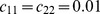, 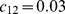 in the correlated case. Numerical (circles) and analytic (solid line) results are compared. **B.** Suppression factor due to correlation, defined as the ratio between the error probability of independent and correlated populations, as a function of the number of neurons, 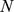; numeric (circles) and analytic (solid line) results. **C.** Error probability as a function of the cross-pool correlation, 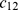, for independent (dashed line) and correlated (circles, 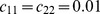) populations; analytic results for correlated population (solid line). 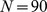. **D.** Error probability as a function of the correlation within Pool 1, 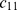, for independent (dashed line) and correlated (circles, 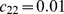, 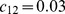) populations; analytic results for correlated population (solid line). 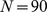. **E.** Probability contours for three examples of neural populations; independent (green cross, 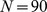, 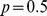, 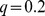), near lock-in correlation (pink dot, 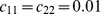, 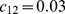), and uneven correlation (blue diamond, 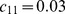, 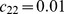, 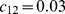). Colored symbols correspond to points on plots in previous panels.

To illustrate this generic mechanism, we have computed the error rate numerically for specific choices of parameters of the firing rates and correlations in the population. (See Materials and Methods for a reminder of the maximum likelihood error and for details on the numerics.) By way of comparison, in an independent population with 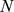 neurons the error rate drops exponentially as a function of 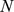 (Fig. 2A). While the error rates for independent and correlated populations start out very similar for small population size, they diverge dramatically as 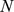 increases to 90 neurons (Fig. 2A). We can define a factor of coding improvement due to correlations as the ratio of the two error rates; this factor exceeds 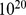 for large populations (Fig. 2B). We can also explore the way in which the error rate changes as we vary the strength of the pairwise correlations at fixed population size. Increasing the strength of correlation across pools, 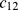, sharply reduces the error rate, while increasing the strength of correlation within pools, 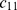 or 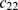, enhances the error rate (Figs. 2C and D).

The important point, here, is that improvements by orders of magnitude do not result from growing the population to unrealistically large numbers of neurons or from boosting the values of pairwise correlations to limiting values close to 1. Correlations may be more or less fine-tuned at a population level, so that the probability of some activity pattern in the population becomes vanishingly small, but no fine-tuning is apparent at the level of *pairwise* correlation. Furthermore, we have focused here on ‘rapid coding’ – situations in which it is not possible to suppress variability by temporal integration. Even then, the massive suppression of error rates occurs in populations of fewer than a hundred neurons and in the presence of realistic correlations ranging from 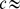 0.01 to 0.03. (Most correlation values reported in the literature have been measured over somewhat longer time scales than the tens of milliseconds of interest here, but see Ref. [21].) Strong error suppression occurs because, even in populations of relatively modest size, weak correlations can significantly deform the shape of the probability distributions of population responses (Fig. 2E).

**Figure 3:**
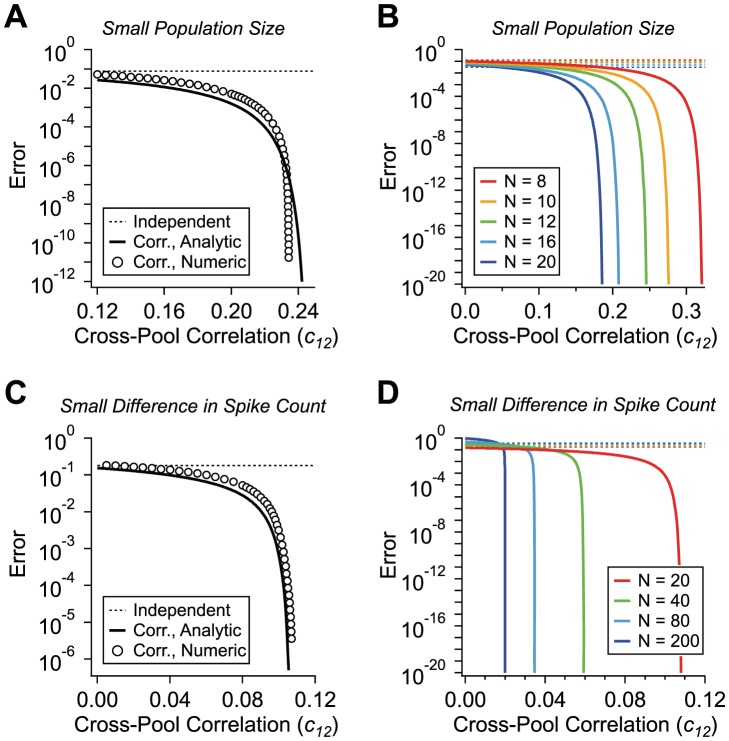
Small correlated populations. **A.** Probability of error as a function of the cross-pool correlation, 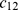, for a small neural population (circles, 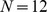 neurons, 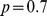, 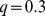, 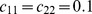), with analytic result for correlated population (solid line) and independent population (dashed line) for the sake of comparison. **B.** Probability of error *versus* 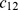 for populations of different sizes (colors); independent population (dashed lines) and analytic results for correlated population (solid lines). **C.** Probability of error versus 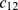 for a neural population with responses differing by an average of 2 spikes (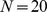 neurons, 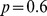, 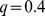, 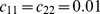); numeric solutions (circles), analytic result (solid line), and independent comparison population (dashed line). **D.** Probability of error versus 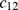 for populations having different sizes but with 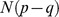 held constant at 2 spikes (colors); independent population (dashed lines) and analytic results for correlated population (solid lines).

In fact, the suppression of the coding error down to negligible values by positive correlation does not even require populations with as many as 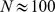 neurons. Such suppression can be obtained in much smaller populations, with a total number of neurons, 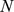, between 8 and 20 and with values of correlations below or not much higher than 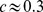 (Figs. 3A and B). Such values of correlations are still well within the experimentally measured range. We also explore another case which, naively, prohibits low-error coding: that in which the firing rates in the two neuron pools differ by very little; specifically, when 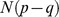 is of order one. This condition implies that the overall activities in a given pool, in response to Target and Distracter respectively, differ by one or a few spikes. In this limiting case, coding with pools of independent neurons fails entirely, with error rates of order one, since the absolute amplitude of fluctuations exceeds unity. In a correlated population, we find, again, a massive suppression of error rates by orders of magnitude, for realistic values of correlation (Figs. 3C and D).

### Analysis of low-error coding

In addition to our direct numerical investigations, we have performed analytic calculations using a Gaussian approximation of the probability distribution (see Materials and Methods for derivations). The analytic results agree very closely with the numeric results (Figs. 2 and 3, solid line vs. circles) and yield simple expressions for the dependence of the error rate upon the parameters of our model, useful for a more precise understanding of the effect of correlation.

The analytic expression of the error rate, 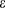, reads 
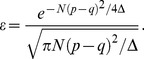
(1)


The numerator in the argument behaves as expected for a population of independent neurons: it yields an exponential decay of the error rate as a function of 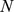, with a sharpness that increases with the difference between 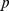 and 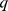. But the denominator,
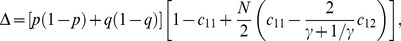
(2)where
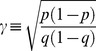
(3)and we have assumed the symmetric case 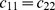 for the sake of simplicity, provides a strong modulation as a function of correlations (Figs. 2 and 3). The quantity in Eq. (2) approaches zero when 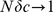, where 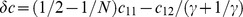. Thus, in a population of tens or hundreds of neurons, it is sufficient that the two terms in 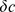 differ by less than a few percent for the coding error to become vanishingly small.

From Eq. (1), it is apparent that the error rate converges rapidly to zero with decreasing 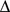, and has an essential singularity at 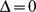. For any well-defined probability distribution, 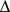 remains non-negative, but it can take arbitrarily small values. When correlations are such that 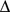 is small enough, we are in a regime of high-fidelity coding. The error vanishes for 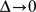; in this limit, the probability distributions corresponding to Target and Distracter are both parallel and infinitely thin. The value of 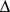 alone does not specify the geometry of the probability distributions entirely; even with 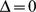, there remain free parameters, namely, the angles along which the elongated distributions lie in the 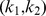 plane (denoted by 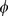 in Fig. 1B). In Materials and Methods, we demonstrate that these additional parameters need not be fine-tuned for high-fidelity coding. In fact, angles can vary by as much as 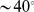 while the error rate remains below 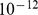.

### Neural diversity is favorable to high-fidelity coding

The simplest version of the 2-Pool model, discussed hitherto, assigns homogeneous firing rate and correlation values within and across each of the two neural sub-populations. Similar homogeneity assumptions are frequent in modeling and data analysis: while response properties vary from neuron to neuron in data, average values are often chosen to represent a population as a whole and to evaluate coding performances. It is legitimate, however, to ask to what extent error rates are shifted in a more realistic setting which includes neural diversity and, in fact, whether high-fidelity coding survives at all in the presence of neuron-to-neuron heterogeneity. We find that not only does it survive but that, in fact, neural diversity further suppresses the error rate.

**Figure 4:**
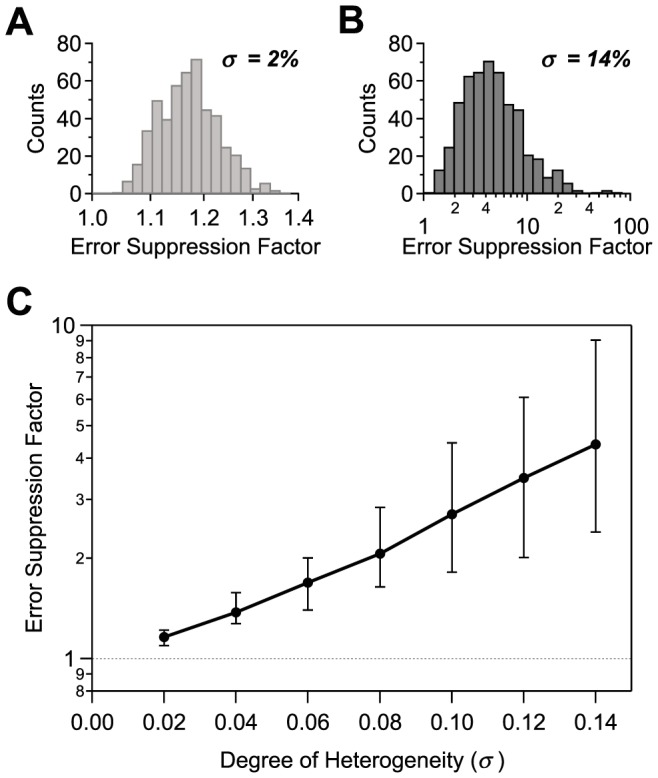
Heterogeneous neural populations. **A, B.** Histogram of the error suppression (error in the homogeneous, 2-Pool model divided by the error in the fully heterogeneous model) for variability values 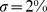 and 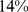, respectively. All suppression values are greater than one. **C.** Value of the error suppression (geometric mean) *versus* the degree of population variability; 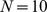 neurons, 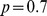, 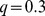, 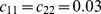, 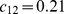. (With these parameters, correlation suppresses the error probability by a factor of 4350 relative to the matched independent population.)

We generalized the 2-Pool model of a correlated population to include neuron-to-neuron diversity, by randomly and independently varying the firing rate and correlation values according to a Gaussian distribution with standard deviation 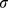, measured as a fraction of the original value. We then computed the error rate in this generalized model and compared it to the corresponding quantity in the homogeneous 2-Pool model. (See Materials and Methods for the precise definition of the heterogeneous model and details on the derivation of error rates.) We found that every single instantiation of neural diversity yielded an improvement in the coding performance (Figs. 4A and B). More diverse neural populations with larger values of 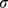 display stronger suppressions of the error rate (Fig 4C). As 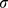 increases, the suppression factor grows both in mean and in skewness, so that a significant fraction of the instantiations of heterogeneity yields a large improvement of the coding performance over the homogeneous case (Figs. 4A *vs.* B).

The degree of error suppression depends, of course, on how much correlation reduces the error relative to the matched independent population in the first place. For the population shown here, neuron-to-neuron variations on a range commonly seen in experiments lead to a suppression of the error rate by a factor of 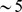 on average and a factor of 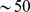 for some instantiations of the heterogeneity (Fig. 4B). These results would differ quantitatively, and may differ qualitatively, in the extreme cases already poised very near lock-in in the absence of neuron-to-neuron variability of the correlation values, as the lock-in limit corresponds to a boundary in the space of covariance matrices.

Above, we have examined the effect of heterogeneity for a simple and contrived case: in a model with homogeneous pools, we have perturbed firing rates and correlation coefficients by small amounts. The results may be different for other forms of heterogeneity. We relegate to a separate publication a more detailed investigation of the quantitative effect of heterogeneity and of the corresponding mechanisms by which coding is improved. We mention, however, that the coding benefit of heterogeneity appears to be a rather general phenomenon [31], [37], [44].

### The mechanism for high-fidelity coding and the ‘lock-in’ phenomenon

The mechanism of dramatic error suppression from positive correlations may be explained in a general manner that does not invoke a specific model or approximation. A powerful description is given in terms of the ‘macroscopic’ variances and covariances of the spike count within and across the two pools: we call 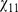 the variance in the spike count, 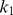, within Pool 1, 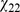 the variance in the spike count, 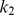, within Pool 2, and 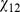 the covariance of spike counts across the two pools. (See Fig. 1B for a visual definition of these quantities, Materials and Methods for mathematical definitions as well as derivations of the results discussed below.)

The variances of the probability distribution of the neural response in the plane 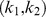 take the form
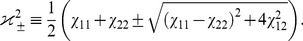
(4)


The angles along which these variances are measured can also be computed similarly (see Materials and Methods). In the case of positive correlation, the angle along which the distribution elongates (*i.e.*, the angle long which 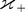 extends, denoted 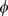 in Fig. 1B) lies between 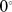 and 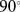. The small variance, 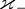, lies at right angle and governs error rate suppression. The smaller 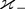 and the more parallel the compressed distributions, the smaller the error rates. The expressions for the variances (above) and the angles (given in Materials and Methods) are general—they do not depend upon the shapes of the distributions or the details of the correlation among neurons—and they give a sense of the extent to which probability distributions of the population response are deformed by correlations. In the specific 2-Pool models we treated above, positive correlations induce massive suppressions of the coding error rate. We expect similar high-fidelity coding whenever the tails of probability distributions fall off sufficiently rapidly.

The limiting case of an infinitely thin distribution occurs when 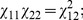
(5)in this case, 
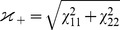
(6)and 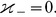
(7)


We refer to Eq. (5) as the ‘lock-in’ condition. When the cross-pool covariance becomes this large, the width of the probability distribution vanishes and the dimensionality of the response space is effectively reduced by one. In the case of homogeneous pools of neurons, we can reformulate this condition using ‘microscopic’ correlations, as 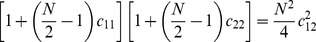
(8)(see Materials and Methods). If the lock-in condition in Eq. (5) (alternatively, Eq. (8)) is satisfied and and _ (alternatively, _ and _) are chosen such as to yield compressed distributions that are parallel, then error rates vanish. (See Discussion for remarks on the nature of the locked-in state.

As we have seen above, even if the cross-pool correlation approaches this lock-in limit without achieving it, still the error rate can be suppressed dramatically. Furthermore, the angles of the two distributions need not be precisely equal. Thus, this amounts to a robust mechanism by which coding and discrimination may be achieved with near-perfect reliability. It does not require fine tuning of the parameters such as the distribution widths and their tilt angles; in particular, we need not limit ourselves to symmetric choices of parameters, as we have done above for the sake of simplicity.

The general arguments presented here also indicate that the ‘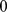 or 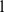 spike’ assumption is inessential and, in fact, that relaxing it may lead to even stronger effects. If individual neurons can fire several spikes in a time window of interest, the code can be combinatorial, but a simple spike count code will do *at least as well* as a more sophisticated combinatorial one. If we stick to the spike count code, the general formulation remains valid. In this situation, allowing many spikes per neurons corresponds effectively to increasing the total number of neurons and, hence, can yield stronger effects for comparable correlation values.

### Correlated populations can code for large sets of stimuli with high fidelity

In most natural situations, the task of organisms is not to tell two stimuli apart but rather to identify an actual stimulus among a wealth of other, possibly occurring stimuli. Visual decoding must be able to assign a given response pattern to one of many probability distributions, with low error. In other words, any pair of probability distributions of neural activity, corresponding to two stimuli among a large set of stimuli, must have little overlap. Thus, the problem of low-error coding of a large set of stimuli amounts to fitting, within the space of neural activity, a large number of probability distributions, while keeping them sufficiently well separated that their overlap be small.

**Figure 5:**
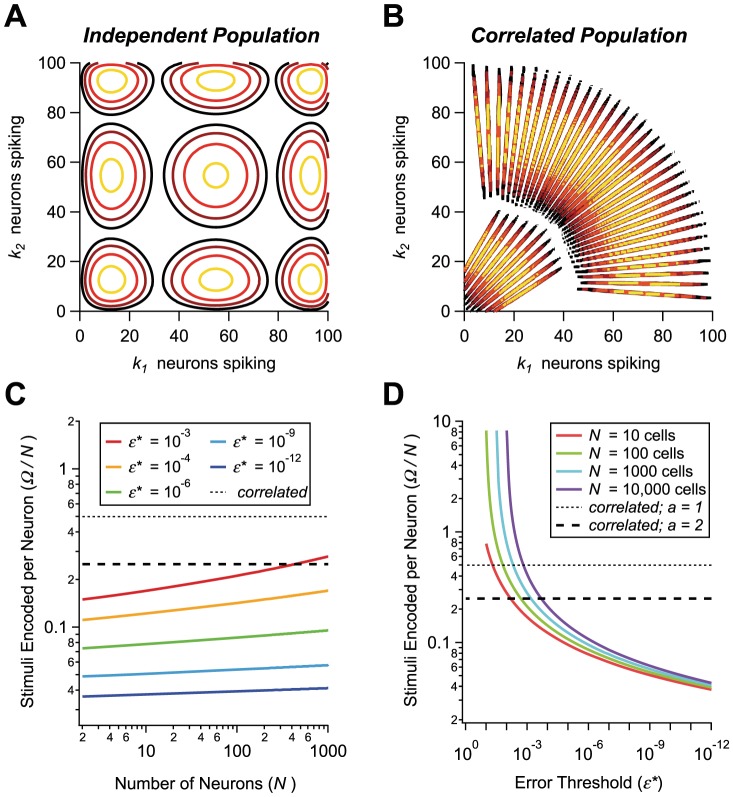
Number of encoded stimuli for independent *versus* correlated populations. **A, B.** Schematics of the optimal arrangement of the probability distributions for independent (A) and correlated (B) populations. Each set of contours represents the log probability distribution of neural activity given a stimulus (hotter colors indicate higher probability). Spacing is set by the criterion that adjacent pairs of distributions have a discrimination error threshold 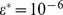. **C.** Number of stimuli encoded at low error, per neuron, *versus* 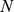, for correlated (thin dashed line for 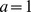, thick dashed line for 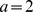) and independent (solid lines) populations, for different values of the error criterion, 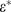 (colors). **D.** Number of encoded stimuli per neuron, for correlated (thin dashed line for 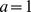, thick dashed line for 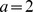) and independent (solid lines) populations, *versus* 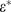, for different values of the number of neurons, 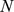 (colors).

It is easy to see pictorially why the presence of correlation is favorable to the solution of this problem. The state of the 2-Pool model is specified by the number of active neurons in Pools 1 and 2, 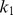 and 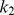 respectively. If neurons are independent, probability distributions (corresponding to different stimuli) have a near-circular shape with variances along the horizontal and the vertical axes of order 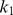 and 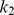 (Fig. 5A). As a result, the only way to prevent tails from overlapping too much is to separate the peaks of the distributions sufficiently. By contrast, since correlated distributions are elongated, their centers can be placed near each other while their tails overlap very little (Fig. 5B). Thus, many more correlated distributions than independent distributions can be packed in a given region in the space of neural responses (Figs. 5A and B).

We call 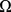 the maximum number of stimuli that a population of neurons can code with an error rate less than 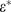 in the discrimination of any stimulus pair. In the case of independent neurons (Fig. 5A), a simple calculation yields 
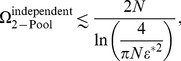
(9)where we have chosen the value of the error threshold to be small enough that 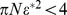 (see Materials and Methods for derivations). In the correlated case (Fig. 5B), distributions are elongated and, provided the correlations values are chosen appropriately, error rates become vanishingly small even if the average firing rates of nearby distributions differ by no more than a few, say 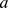, spikes. We then obtain 
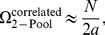
(10)since distribution centers can be arranged along a line that cuts through the space of responses—a square with side 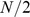 in the positive 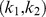 quadrant. (Note that more than one row of distributions may be fitted into the response space of the neural populations if the distributions are not too broad in their elongated direction, with a resulting enhancement of 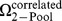. Figure 5B illustrates a case in which three rows are accommodated. We do not include these extra encoded stimuli in our calculations, thus remaining more conservative in our estimate of coding capacity.) According to our earlier results (Fig. 3D), even in moderately small populations the error rate becomes exceedingly small for realistic choices of the correlation values when the distribution centers are two spikes away from each other. Thus, we can choose the value 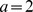 to obtain an estimate of 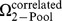. Putting all this together, find that for low enough 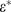 correlated coding always wins over independent coding (Fig. 5C) because 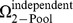 depends upon 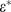 much more strongly than 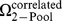 does. Furthermore, in the uncorrelated case and in the limit of small error thresholds, increasing the population size yields only a negligible enhancement of the number of faithfully encoded stimuli, 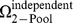, because this quantity is largely insensitive to the size of the population (Figs. 5D).

### Positive correlations in a diverse neural population can enhance capacity by orders of magnitude

Our arguments suggest that we ought to examine the behavior of the capacity of heterogeneous neural populations because a greater degree of heterogeneity amounts to higher dimensional versions of the situations depicted in Figs. 5A and B, as we explain now. We define the 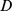-Pool model: a heterogeneous generalization of the 2-Pool model in which the neural population is divided into 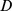 sub-populations. As before, firing rates and correlations are homogeneous within each pool and across pool pairs. For the sake of simplicity, we consider symmetric pools with 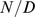 neurons each; we also expect this arrangement to be optimal for coding. The state of the model is completely defined by the number of active neurons in each pool.

**Figure 6:**
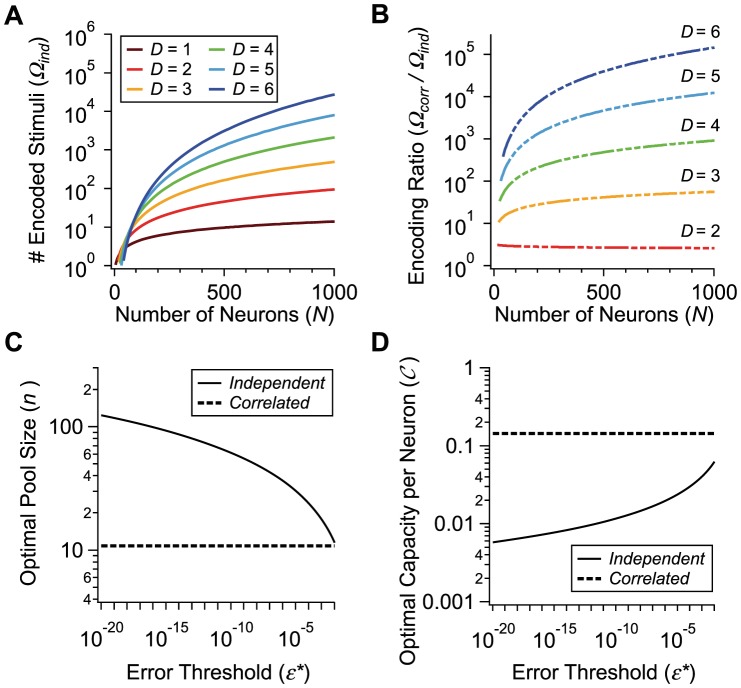
Coding capacity of heterogeneous populations. **A.** Number of encoded stimuli *versus* 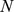, for an independent population divided into different numbers of pools, 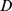 (colors); the error criterion is 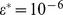. **B.** Ratio of the number of encoded stimuli in a correlated population and the number of encoded stimuli in a matched independent population, for different numbers of pools 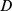 (colors). **C.** Optimal pool size, 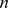, *versus* error criterion, 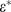, for correlated (dashed line, 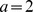) and independent (solid line) populations. **D.** Optimal capacity per neuron, 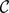, *versus* error criterion, 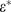, for correlated (dashed line, 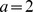) and independent (solid line) populations.

In order to estimate 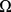, we have to examine how probability distributions corresponding to different stimuli can be fitted within a 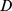-dimensional box enclosing 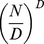 neural states. And overlaps among distributions have to respect the prescribed error rate threshold. In the case of independent neurons we have to fit in 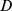-dimensional near-circular objects, whereas in the case of correlated neurons we have to fit in slender objects. It is intuitive that it is easier to pack cucumbers in a box than to pack melons of a comparable volume, because a greater amount of empty space is wasted in the case of spherical objects such as melons, and indeed we find here that a greater number of correlated distributions, as compared to independent distributions, can be packed in the space of responses. The calculation gives
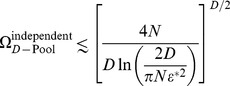
(11)(Fig. 6A, see Materials and Methods for derivations). Notice that the number of possible stimuli encoded by the independent population increases for greater heterogeneity (larger 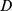).

In the case of correlated neurons, distributions may be compressed along one, two,…, or 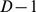 directions, by tuning one, two,…, or 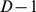 effective parameters, respectively, in such a way that the matrix of covariances come with one, two,…, or 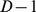 near-vanishing eigenvalues. In the latter case, indeed the most favorable scenario, we have to pack near-one-dimensional objects. As before in the case of a two-pool population, we can assume that neighboring distributions centers are separated by 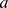 spikes, and we obtain
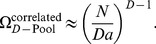
(12)


This simple result follows from the observation that distribution centers can be arranged on a hyperplane that cuts through the hypercube of the space of responses (see Materials and Methods for a more detailed discussion and a slightly more careful bound). From these expressions we can conclude that the enhancement in capacity due to correlation is significant, and that the enhancement increases with the degree of heterogeneity (Fig. 6B).

The number of stimuli encoded with tolerable error rate, 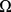, scales differently with model parameters in the independent and correlated cases. In order to focus on this scaling behavior, we define the ‘capacity per neuron’, 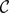, by analogy to the information conveyed by each neuron in a population of perfectly deterministic neurons. In the latter case, the population has access to 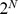 response patterns that can code for stimuli with perfect reliability. Each neuron conveys 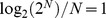 bit of information. Consequently, we define the capacity per neuron as
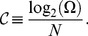
(13)


It is a measure of the mutual (Shannon) information per neuron in the population in the limit of very small 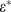.

To explore the scaling behavior of correlated *versus* independent populations, it is reasonable to ask what degree of heterogeneity, as measured by 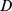, maximizes 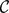 for each value of 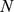. Equivalently, we can ask what pool size, 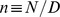, maximizes 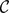 (Fig. 6C, see Materials and Methods). In the correlated case, the optimal capacity obtains when heterogeneity is strong, in fact so strong that the number of neurons per pool, 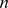, is as small as 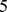 to 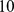 neurons for the choice 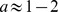. From the optimal pool size, we find that the optimal value of the capacity per neuron is given by
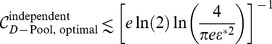
(14)and
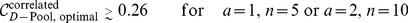
(15)in the independent and correlated cases respectively (see Materials and Methods for derivations). The independent capacity becomes very small at low-error thresholds, while the correlated capacity remains fixed and in fact of the same order as the capacity of a perfectly reliable neuron (Fig. 6D). Thus, in the limit of low error, the capacity and hence information encoded per neuron exceeds the corresponding quantity in an independent population by more than a factor of 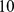. By comparison, one often finds analogous effects measured in a few percent in other studies.

We have put forth the following picture. For a neural population to code for a large set of inputs reliably, it breaks up into small pools with about ten neurons, with correlation across pools stronger than correlation within pools. These pools are small enough that their number is large, and consequently the response space is high-dimensional. But, at the same time, the pools are large enough that realistic correlations lock them in and yield effectively lower-dimensional response distributions. In a sense, a pool behaves like a ‘deterministic meta-neuron’ which obeys a near-digital code. In the 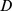-dimensional space of population activity, variability is confined to one (or more) directions. In the extreme case in which the population responses for different stimuli differ by no more than one or two spikes (as illustrated in Fig. 5B), the orthogonal 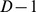 (or fewer) directions are relieved from variability and the code is near-digital in that sub-space. Clearly, this represents the most extreme case of high-fidelity coding; even away from this limit, when there is a degree of variability along all directions, correlation can significantly enhance capacity. We emphasize, also, that, even in the limiting case, the suppression of the variability can be checked only in simultaneous measurements of at least all the neurons in a given pool; measurements of, e.g., pairs of neurons will yield as much variability as if neurons were independent.

### Experimental test of favorable correlations

**Figure 7:**
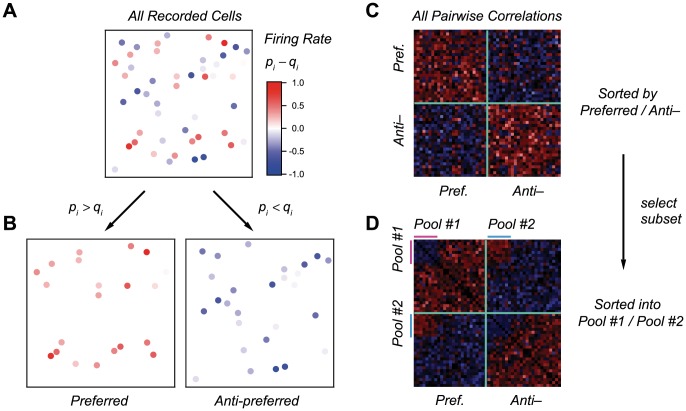
Schematics of an experimental test of high-fidelity correlated coding. **A.** Representation of a population of 50 neurons recorded under two stimulus conditions. Each cell displays firing rates 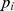 and 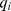 in response to the two stimuli, respectively; the color scale shows the difference in rates, 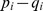. **B.** The population is divided into two groups, depending on whether their cells fire more significantly in response to the first (preferred) or the second (anti-preferred) stimulus. **C.** Matrix of correlation values among all pairs of neurons (red  =  large, blue  =  small, black  =  average), divided into preferred and anti-preferred groups. Although the overall correlation is stronger for neurons with the same stimulus tuning (average correlation of pref-pref  = 0.206, anti-anti  = 0.217, and pref-anti  = 0.111), a subset of neurons (Pool 1 and Pool 2) are identified which have the pattern of correlation favorable to lock-in. **D.** Matrix of pairwise correlations after re-labeling cells in order to sort out Pools 1 and 2. Now the favorable pattern of correlation is visible.

If neural populations rely upon correlation to achieve high-fidelity coding, we expect that patterns of correlations resembling those postulated in our model can be found in data. Namely, our hypothesis predicts that subsets of similarly tuned pools of neurons will exhibit weaker within-pool correlations than cross-pool correlations. In order to check this prediction, the response of a neural population to a pair of stimuli or a pair of stimulus classes has to be recorded (Fig. 7A). This population is divided into a group of cells that fire more strongly to the first stimulus and the rest that fire more strongly to the second stimulus (Fig. 7B). Note that this step is always possible and that all cells can be thus assigned.

Next, one would have to identify pools of neurons, within the population, such that the correlations relative to these pools are near lock-in. But this is a stringent requirement, which would involve exceedingly heavy numerical processing. Instead, one can search for subsets of the population that have stronger correlation across the groups than within (Fig. 7C), as this is a definite requirement in the proposed scenario—and the one that may appear counter-intuitive. For recordings with several tens of cells, there is a very large number of possible subsets, so an exhaustive search may not be feasible. Instead, there exist a number of faster search strategies. For instance, one can score each cell according to the sum of its pairwise correlation to all cells in the other group minus the sum to all cells within its stimulus-tuned group. This yields a rank ordering of cells, which can be used for selecting favorable subsets. In addition, searches can be made iteratively, starting with 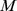 cells and finding the best next cell to add to the subset. Once a subset is identified, a quick assessment of the role of correlation can be made using average firing rates and correlations to calculate the error rate in the Gaussian approximation (Eq. (1)). As seen in Figs. 2 and 3, this approximation is highly accurate. Then, for the most favorable subsets, a maximum entropy calculation can be carried out to estimate the discrimination error taking into account the true experimentally observed heterogeneity. As indicated by Fig. 4, the homogeneous approximation is not only quite close to the real error rate, but it also serves as an upper bound on the error. In this manner, subsets of neurons with correlation patterns favorable to lock-in can be identified in neurophysiological recordings.

## Discussion

### Summary

We have shown that a class of patterns of positive correlation can suppress *coding errors* in a two-alternative discrimination task (Figs. 2A and B). The idea that correlations among neurons may be favorable to coding was noted earlier. What is new, here, is the demonstration of the extreme degree of the enhancement in coding fidelity from positive correlation — several orders of magnitude rather than a few tens of a percent. Furthermore, this generic result does not require unrealistic values of correlation or population size: it can operate at the moderate values of correlations recorded experimentally (Figs. 2C and D) and in populations with as few as 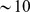 neurons (Figs. 3A and B). In fact, massive error suppression may occur even when average activities in a neural pool in response to different stimuli differ by one or a few spikes (Figs. 3C and D)—a limiting, but realistic, situation in which coding with independent neurons fails completely.

We have also shown that correlations can boost dramatically the *capacity* of a neural population, *i.e.*, the number of stimuli that can be discriminated with low error (Figs. 5 and 6). For independent neurons, the mean firing rates of the population in response to different stimuli must differ by a substantial amount to allow low error, because the firing variability about the mean is not harnessed by correlation. By contrast, in the presence of correlation, neural response distributions can deform into slender objects, effectively lower-dimensional objects, which can be fitted much more efficiently within the population's response space (Fig. 5B).

At lock-in, response distributions become strictly lower-dimensional (one-dimensional in the extreme case); in this limiting case, small pools of neurons within the population behave like ‘deterministic meta-neurons’ which obey a near-digital code. While our calculations have focused on this extreme limit of ‘lock-in’, the brain need not achieve it strictly. The logic is that if this upper bound is insignificant then one can rule out this coding scheme, but that if the upper bound is highly significant – as we show here – then it is more plausible that the brain might find beneficial adaptations to make use of the mechanism. We note that, even in the lock-in limit, it is not possible to read off the high reliability of the population response from ‘local’ quantities such as pairwise correlation coefficients. The latter display as much variability as in the case of independent neurons. High-fidelity coding is a collective phenomenon.

Furthermore, we have demonstrated that diversity in neuron-to-neuron response, and more generally heterogeneity of the population response, further enhances the effect of correlation (Fig. 4 and Figs. 6A and B). Indeed, the advantageous role of heterogeneity seems to be a rather general feature of population coding, and it has been illustrated within various approaches [31], [37], [44]. We refer to the phenomenon in which neural correlation suppresses the discrimination errors to negligible values and dramatically boosts the capacity of a population as *high-fidelity coding*. In passing, we note that high-fidelity coding does not, in principle, require equal-time correlation: the same mechanism can be at play when the correlations that matter involve different time bins, such as in ‘spike-latency codes’ [45]. Finally, we have proposed a possible analysis of neural data that aims at uncovering favorable patterns of correlation (Fig. 7).

### How extreme is lock-in?

We showed that, in the limit of large enough populations or strong enough pairwise correlations, the distribution of activity of the population can ‘lock in’ to a state of lower dimensionality. While, in this state, *macroscopic* correlations (among spike counts in sub-populations) reach limiting values, one may wonder about the nature of the *microscopic* correlations. Furthermore, we can ask how finely the parameters of the model ought to be tuned for a significant effect on coding to obtain.

The positivity of probability implies constraints upon moments of the neural activity; in particular, we have 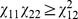. This bound is achieved by the lock-in condition given in Eq. (5). Thus, lock-in embodies the limiting case of maximum macroscopic correlation between Pools 1 and 2, but there remains a significant amount of (microscopic) variability even at lock-in. The specificity of lock-in is that it forbids a subset of the microscopic patterns, i.e., that these occur with vanishing probability. However, at lock-in the system is not confined to a single output pattern. A large set of patterns can occur with non-negligible probability each—hence the variability—and the remaining patterns are ruled out—hence the vanishing overlaps and error rates.

In fact, generically, positive correlations will enhance the marginals of the probability distributions of activity patterns. For example, the variability in a given pool will be boosted by correlations. Thus, for measurements within a given pool, responses will appear *more* variable than independent ones, even at the lock-in limit. Furthermore, while the distribution of population states reaches a singular limit at lock-in, this cannot be read off from individual measurements of pairwise correlations (even is the pair of neuron straddles two different pools). While macroscopic correlation coefficients have been pushed to their limiting values at lock-in, microscopic correlation coefficients remain moderate (and well below any limit one would obtain by considering pairs or small groups of neurons).

In the Gaussian approximation of the two-pool model, only patterns with a fixed ratio between 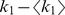 and 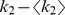 are allowed at lock-in. In the absence of correlations, allowed output patterns fill a two-dimensional space—the 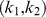 plane. When correlations push the system to lock-in, output patterns are confined to a one-dimensional space—the 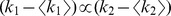 line. This dimensionality reduction results in error rate suppression and in increased capacity. In the higher-dimensional case of full heterogeneity (and Gaussian variability), the question of lock-in amounts to asking whether one or several eigenvalues of the covariance matrix become vanishingly small. A population attains the actual lock-in state only for specific values of pairwise correlation and firing rate; however, we have shown that the error rate can reach near-vanishing values for a range of parameters that do not bring the population all the way to the lock-in condition. This result on robustness is generic as it relies only upon the rapid fall-off of the tails of the response probability distribution.

This points to the second question we posed above, namely, to what extent are the parameters in the model fine-tuned. Clearly, in order to reach the singular, lock-in limit, an effective parameter—a combination of firing rates and correlation coefficients—has to be fine-tuned. There are, however, two important points to note. First, as mentioned above, astronomical enhancement of coding performance occurs *near* lock-in already; there is no need to be *at* lock-in. Second, while one effective parameter ought to be fine-tuned, others do not have to be. In Materials and Methods, we provide a detailed study of the coding performance as a function of variations in these additional parameters, and we demonstrate that the enhancement of performance is highly robust to parameter perturbations.

Finally, we point out an important distinction, which may play a major role in the issue of fine-tuning. Throughout, we have been referring to ‘parameters’ when discussing the response properties, i.e., firing rates, correlation coefficients, and combinations of these. But, in reality, ‘neural processing parameters’ or ‘biophysical parameters’ (such as temporal filters, non-linear transfer functions, synaptic weights, etc) are the ones which are putatively tuned, in an actual brain area. Ultimately, one would like to know to what extent fine-tuning is stringent in the space of these parameters. While a detailed answer to this question certainly lies beyond the scope of the present paper, we can offer a preliminary comment. Intuition as well as exploratory numerical work indicate that, in the space of the ‘biophysical parameters’, rather than fine-tuning parameters, what will matter for a high coding performance is that some parameters be sufficiently strong (e.g., synaptic weights sufficiently large to build up significant correlation). Thus, while high-fidelity coding may require (a relatively) fine tuning in the space of ‘correlation parameters’, fine-tuning is not necessarily required in the space of the ‘biophysical parameters’.

### Relation with earlier work on coding with correlated neurons

A number of theoretical studies have explored the role of correlation in neural coding, with the use of different neuron models and information theoretic measures [25], [26], [27], [28], [29], [30], [31], [32], [33], [34], [35], [36], [37], [38], [39], [40], [41], [42]. If response properties are homogeneous among neurons, positive correlation is detrimental to coding: it tends to induce neurons to behave alike, and thereby suppresses the advantage of coding with a population rather than with a single cell (see Text S1, Supplementary discussion for detailed arguments). By contrast, if response properties vary among neurons, positive correlation can be either unfavorable or favorable [28], [29], [30], [31], [34], [39], [37], [41], [42]. Put more generally, when the scale of correlation is comparable to that of the informative mode in the system (dictated, *e.g.*, by the response tuning curve), then correlation enhances the confounding effect of noise (see Text S1, Supplementary discussion for a simple illustration of this mechanism). But when the scale and structure of correlation is very different — as in the case of uniform positive correlations, in the case of negative correlations (anti-correlations), or in models with heterogeneity — correlation can relegate noise to a non-informative mode [28], [30], [31], [41], [42]. (We recall that we are focusing exclusively upon the case of stimulus-independent covariance matrices and, hence, stimulus-independent pairwise correlations. Experiments indicate the presence of both stimulus-independent and stimulus-dependent correlations.)

In the case of stimulus-independent, positive correlation, earlier studies have formulated a mechanism by which correlation can relegate noise to non-informative models and, hence, enhance coding fidelity [25], [27], [28], [46], [30], [31], [32], [38], [39], [41], [42]. Namely, that negative signal correlations (anti-correlations) should be supplemented with positive noise correlations. To be explicit, this means that when neurons respond differentially to different stimuli, on average, then the variability about this average response should be correlated positively; this mechanism is illustrated in Fig. 1B and sets the starting point of our study. Conversely, negative correlations (anti-correlations) are favorable in the case of positive signal correlation. These statements have been established following different routes in the literature. They can be read off in full generality, that is, without invoking any particular neuron model or form of the neural response, from the expression of the mutual (Shannon) information [29], [33], [34], [39]. This is done by rewriting the mutual information in a form that displays contributions from firing rates, correlations, and the interplay of firing rate and correlation patterns. Approaches using the mutual information have the merit of elegance and generality. However, for quantitative estimates they require the implementation of specific response models; furthermore, they are difficult to apply to large populations of neurons because of sampling limitations and mathematical difficulties.

Similar results can be derived from the form of the Fisher information [28],[30],[31],[39],[41],[42], often used to establish bounds on the estimation variability in the case of continuous stimuli. Most studies consider neurons with broad tuning properties and find that positive correlations are unfavorable if they decay on the scale of the tuning curve. Positive correlations were observed to be favorable in cases in which they are uniform among all neurons or have a non-monotonic profile according to which similarly tuned neurons are less correlated than neurons that differ greatly in their tuning. In all cases, however, positive correlation enhanced the coding fidelity by modest amounts. In the next section, we discuss these quantitative aspects in greater detail, as well as their correspondence with our formulation and results.

In models of broadly tuned neurons with uniform pairwise correlation over the entire population, coding becomes increasingly reliable as the quantity 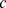 tends to 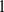. For example, the Fisher information is boosted by a factor 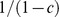 as compared to the case of independent neurons [28]. Thus, strong correlation-induced improvement in coding performance occurs only in the unrealistic limit of 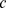 close to 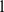. The situation is different in our simple models. There, high-fidelity coding requires that the modified quantity 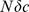 approach 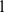, where 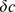 is a weighted difference of cross-pool correlation values and within-pool values, be small (see, e.g., Eqs. (2)). The presence of similarly tuned pools of neurons, within the population, amplifies the effect of weak pairwise correlation to produce profound changes in the activity patterns of the neural population. Since correlation values are in the range 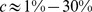, values of 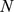 as modest as a few tens or a few hundreds are sufficient to bring the quantity of interest, 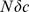, extremely close to 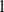.

Similarly, Ref. [30] showed that coding can be enhanced by a large factor in the presence of anti-correlations as weak as 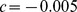 (as quoted, also, in Ref. [39]) and Refs. [37], [41] reported significant boosts of the Fisher information of positively correlated neurons in the presence of heterogeneous tuning functions. This occurs for populations with hundreds of neurons and it is yet another illustration of the significant effect that can take place when 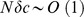. In the present work, we have shown that similarly large effects can occur due to the experimentally more typical positive correlations, and in the context of much smaller neural population with no more than a few tens of neurons.

We remark in passing that there are other mechanisms by which confounding noise can be relegated to non-informative dimensions. In the context of broadly-tuned neurons and long-range correlation—the usual setup of studies which make use of Fisher information—the presence of neuron-to-neuron variability (e.g., in the firing rates) can do the trick [31], [37], [41], [42]. In the absence of variability, positive correlation suppresses the coding performance as compared with an independent population. Neuron-to-neuron variability introduces a new dimension, namely, modulations much finer-grained than the scale of tuning and correlation, in which information is stored. Then, in a correlated population one retrieves, roughly, the coding performance of an independent population. This mechanism cannot, to our knowledge, generate substantial improvement in coding performance over that of an independent population.

A separate line of investigation of the properties of coding in the presence of correlation focuses upon ‘interactions’ (parameters of the probability distribution of population activity) instead of correlation coefficients as its central objects [47], [48], [49]. When a maximization procedure is applied to the mutual information between the distribution of parameters and that of population activity, in a noisy regime one obtains positive interactions and, correspondingly, positive correlation, which enhance the encoded information appreciably compared to an independent population [50]. We note that, there, and at odds with the case we studied here, correlations depend upon the stimulus since some parameters are stimulus-dependent.

### Quantitative comparisons among information theoretic measures

As mentioned in the introduction and in the previous section, earlier investigations which exhibit an improvement of the coding performance due to positive correlation find that the latter is rather limited quantitatively. Specifically, the Shannon information or the Fisher information (depending on the study) in the correlated population exceed that in the equivalent independent population by less than a factor of 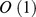. As stated above, the Fisher information can be boosted by a factor 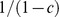 as compared to its counterpart for a population of independent neurons; for typical choices of correlation values, this yields an improvement of 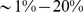. By contrast, in the present study we claim that positive correlation can enhance coding fidelity by massive factors, and that this effect can exist even in small populations of neurons. But how are we to compare our results to earlier results, since the former are expressed in terms of error rate and capacity while the latter are expressed in terms of information measures?

In the case of an unbiased estimator, the Fisher information, 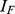, bounds from below the discrimination error, 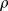, of a continuously variable stimulus: 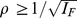 [51]. Thus, if the stimulus spans a space of size 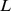 then the number of stimuli that can be distinguished reliably is calculated as
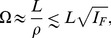
(16)so that the capacity per neuron scales with the Fisher information as 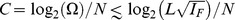. (A rigorous version of this result was derived for a population of independent neurons in Refs. [52], [53].) If correlation enhances the Fisher information by a factor 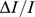, 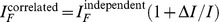, then the number of distinguishable stimuli is correspondingly enhanced according to 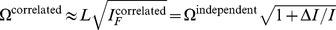. Thus, we have
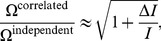
(17)and
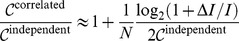
(18)or
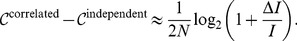
(19)


We can now relate the earlier results in terms of Fisher information to our results in terms of capacity through these formulæ.

An enhancement of the Fisher information given by 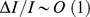 or, to be more specific, 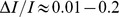 as suggested by earlier theoretical studies, amounts to a small increase of the number of distinguishable stimuli by a factor 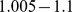. Similarly, the difference between correlated and independent capacity per neuron decays inversely proportionately with 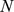; in a large population, the improvement becomes negligible. By contrast, we found that the ratio 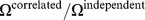 can attain large values (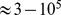, Fig. 6B) and that the difference between the correlated capacity per neuron, 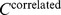, and the independent capacity per neuron, 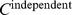, can be significant (Fig. 6D). In brief, earlier studies have demonstrated that, in spite of positive correlations, coding can be as efficient as in an independent population or even slightly better. Here, we show that, provided true population effects are taken into account, positive correlation can have a profound quantitative effect in that they can modulate the way coding measures scale with the number of neurons in the population and, as a result, yield a massive enhancement in coding fidelity.

To conclude the comparison among information measures, we note that, for continuous stimuli, the Fisher information is a natural performance metric. In this case, stimulus entropy always exceeds that of the population response, and the estimation variability decreases with population size, so that one is interested in quantifying the precision of estimation in the large-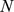 limit. By contrast, here we treat the case of a discrete stimulus, where the entropy is small and discrimination can be achieved with great reliability. This regime is clearly relevant to tasks like decision-making, language, and abstract thought: each categorization error imposes a cost on the organism, making it relevant to characterize coding performance using the error rate rather than the mutual information. Much of computational neuroscience work devoted to networks of neuron has focused upon large-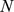 situations. The regime at hand here is somewhat new in character: the largest number is not 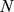, the population size, but rather 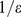, the inverse discrimination error. In fact, a number of neurons as small as 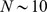 can achieve inverse error rate, 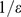, several orders of magnitude larger. Given the breadth and accuracy of cerebral function, and the brain's limited size, we expect this regime to be relevant to diverse instances of neural processing.

### Relation with recorded cortical data

A detailed analysis of neurophysiological data must await a subsequent study. Here, we mention several observations which are consistent with our experimental prediction. Patterns of correlations with stronger cross-pool values may at first seem unlikely; this intuition comes mainly from our knowledge of the primary visual cortex and area MT, in which neurons with similar orientation tuning or directional preference are more strongly correlated, on average. But recent results in the literature hint to the fact that inverse patterns of correlation, with stronger cross-pool values, may well be present in the brain and favorable to coding. Romo and colleagues have reported precisely this phenomenon in S2 cortex: in some fraction of their data (but not in others) they found positive correlation among pairs of neurons with opposite frequency-tuning curves [32]. This pattern of correlation resulted in an improvement in the threshold for discrimination between different frequencies of tactile stimulation. Maynard et al. similarly found that a model that incorporated correlation reduced discrimination errors, as compared to an independent model, for groups of up to 16 cells in M1 during a reaching task [54]. Here, correlations elongated the response distributions precisely in the manner depicted in Fig. 2B. Interestingly, Cohen and Newsome observed that MT neurons with widely different direction preferences displayed stronger positive noise correlation when the discrimination task was designed in such a way that, effectively, they belonged to different stimulus-tuned pools [55]. In another cortical study, Poort and Roelfsema demonstrated that noise correlation can improve coding between V1 cells with different tuning, partially canceling its negative effect on cells with similar tuning [56]. Finally, Gutnisky and Dragoi [57] observed that after rapid (400 ms) adaptation to a static grating, pairwise correlation coefficients among neurons with similar tuning decreased more than for neurons with somewhat different tuning preferences — a trend in adaptation which agrees with the proposed favorable pattern of correlation. However, we note that correlation among neurons with very different tuning preferences also dropped after adaptation, so that the trend may be mixed.

### Read-out and decoding from correlated neurons

In this paper, we have been concerned with establishing bounds on the information that can be extracted from a population of correlated neurons, by calculating the error rate of an optimal deterministic decoder and by estimating the encoding capacity of the population. A separate question is: How do actual, read-out neural circuits ‘decode’ the information contained in the activity of a correlated population? While this question is a very interesting one, which, quite generally, pertains to almost all studies of the neural code, it is also a difficult one because of a biological issue and a conceptual issue. The biological issue is that we don't yet know enough about the constraints that apply to decoding: the architecture of read-out circuits, the relevant biophysical properties of read-out neurons, etc. The conceptual issue is that we don't know in what form the information is decoded: even if, ultimately, the information is represented by some kind of ‘grand-mother cell’, the latter may result from many layers of processing. Thus, decoding circuits may be highly non-trivial.

There are many examples in the literature in which decoding is discussed in the context of a one- or two-layer perceptron-like read-out model. The motivation for such models is that they can be implemented accurately by the known, basic properties of neurons; hence, they make simple and likely candidates for actual read-out networks. Here, we illustrate a similar model devised as a decoder from a correlated population.

**Figure 8:**
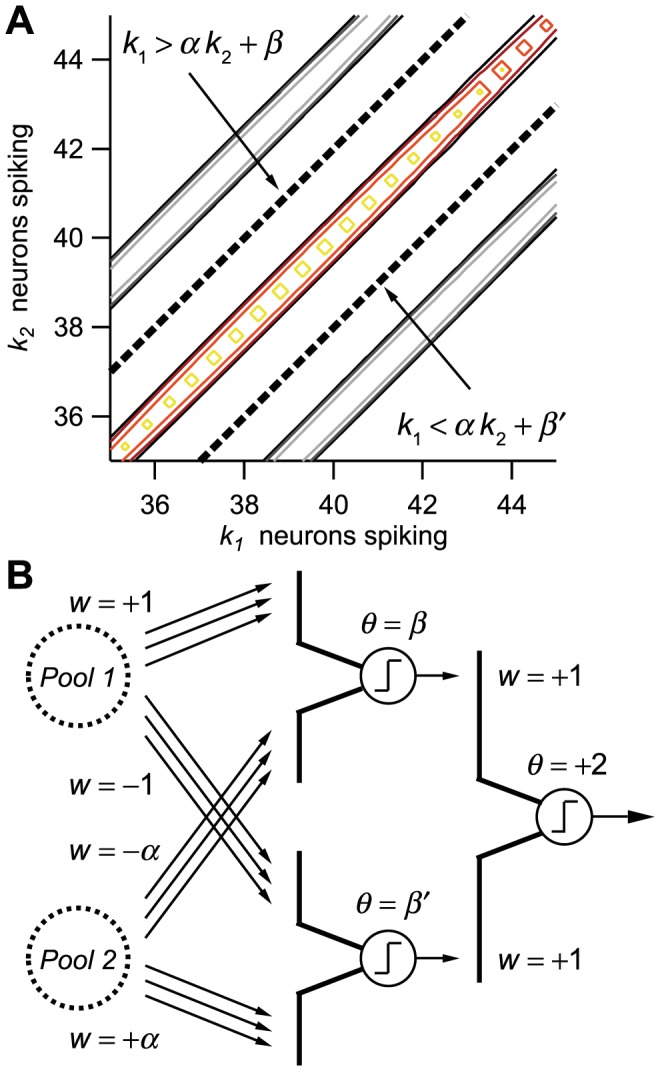
Illustration of a proposed decoding mechanism and circuit. **A.** The decoding mechanism is illustrated in the case of a two-pool model, in which 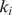 denotes the spike count in Pool 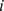. The stimulus to be decoded elicits the distribution of activities represented by the yellow-red contour lines; other distributions, in blue-grey, flank it and result from different stimuli. Optimal decision boundaries (dashed lines), defined by simple inequalities, are implemented by the read-out circuit. **B.** The read-out circuit is a two-layer perceptron. In its first layer, excitatory and inhibitory inputs from both pools are non-linearly summed into two intermediary read-out neurons; the synaptic weights and thresholds (equivalently, baselines) are chosen such that the two intermediary neurons implement the inequalities 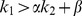 and 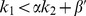, respectively. Their two outputs are then summed non-linearly in turn, so that the ‘decoder neuron’ is active only if both inequalities are satisfied.

The read-out circuit ought to implement the optimal decision boundaries. We focus, first, on a two-pool model of correlated population. In the simplest case with symmetric parameters (Fig. 1), the decision boundary is given by 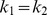, where 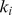 is the spike count in Pool 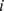. In the case of non-symmetric choices of parameters, the decision boundary becomes 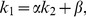
(20)where 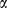 and 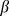 are constants. In the presence of many stimuli, the decision boundaries between pairs of stimuli are given by Eq. (20) with different values of the constant 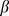 for different stimulus pairs (Fig. 8A). (For stimulus-independent correlation, the constant 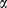 is fixed.) Thus, if 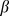 and 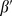 correspond to two ‘neighboring’ decision boundaries, then the intervening stimulus is uniquely identifies if both inequalities 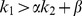 and 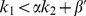 are satisfied (Fig. 8A). The task of a ‘decoder neuron’ is to be active when both these inequalities are satisfied and inactive otherwise: its activity then represents the presence of a given stimulus. This is achieved trivially by a two-layer perceptron in which excitatory and inhibitory inputs from Pool 1 and Pool 2 are summed non-linearly (Fig. 8B). The constants 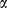 and 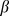 are implemented by the strengths of the synapses and the value of the perceptron threshold (equivalently, baseline), respectively.

In the case of a many-pool model of correlated population, the decoding rule is, conceptually, the same, but is calculationally more involved as it is carried out in a higher-dimensional space. In the case of 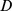 homogeneous pools of correlated neurons, and 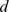 dimensions along which the probability distributions are ‘compressed’ (in our examples above, we had chosen 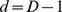), a given stimulus is identified by 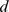 pairs of inequalities analog to the above ones. In other words, to identify a given stimuli, the decoder has to carry out at most 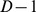 pairs of binary decisions.

A few comments are in order, here, about this model of decoding. First, we note that this simple perceptron read-out achieves optimal decoding, as it implements the optimal decision boundaries (up to processing noise). Second, we point out that the complexity of the proposed decoder is comparable to that of a decoder from independent neurons; thus, the presence of correlations does not render decoding more problematic. Third, we emphasize that, when reading out many stimuli from a given, correlated population, the decoder cells do not need to collect their inputs from different sub-divisions of the population, nor do different arrays of synaptic weights need be learned for each stimulus. Fourth, and finally, we mention that in the more realistic case of a heterogeneous neural population, an optimal decoder would have to implement a non-linear decision boundary (instead of the linear ones illustrated in Fig. 8A). As a result, the read-out circuit would be more involved. However, one might still be able to recover nearly optimal performance with simpler decoders if the heterogeneity is not too severe.

### Sensory coding requires extremely low error rates

Everyday vision occurs in a different regime than that probed in many of the classic studies in visual psychophysics. Our retina is presented with complicated scenes in rapid succession—either because of saccadic eye movements or because of motion in the scene itself—from an enormous set of possibilities. Often, we seek to recognize the presence of a target stimulus or stimulus class and distinguish it from every other possible stimulus. For example, we might want to recognize a friend's face in a particular spatial location. That location might contain another person's face, or a flower, or myriad other objects, which we do not want to mistake for our friend's face. Alternatively, the target stimulus is often a class of related stimuli, such as that friend's face from a variety of angles or the presence of any human face, so that a class of visual patterns on the retina, rather than a single fixed pattern, is to be identified.

In this regime, one distinguishes two kinds of coding error: *misses* and *false alarms*. In the former, one does not pick up on the target stimulus; in the latter, an absent target stimulus is erroneously perceived. While both kinds of error take place occasionally (think of mistaking a wavy tree branch for a snake, as a false alarm), the effortless feat of the visual system in avoiding them most of the time is rather bewildering. If we pause a moment on what this feat means at the neural level, as illustrated by the following example, we realize that it requires extremely precise coding.

Imagine stretching out on your hotel bed in a tropical country. If there were a very large spider on the ceiling, you most likely would want to detect it and detect it promptly. For the sake of concreteness, let us imagine that the spider has a size of three centimeters and is three meters away, subtending a visual angle of 0.01 radians. Thus, there are 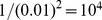 possible spider locations on the ceiling. If you are able to detect the spider in any of these locations, it implies that your brain must effectively have a ‘spider-detector’ circuit that reads out activity from a retinal population that subtends each of these spatial locations. If you would like to detect the spider quickly, say in 100 milliseconds, then there are 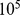 possible spider-detection events per second. Now, if each detector operates at a false alarm rate that would naively seem low enough to be acceptable, say 0.001—i.e., a probability of error of a tenth of a percent— you would still perceive 100 virtual spiders per second! If we impose the additonal cautionary constraint that spider detection be possible only within the parafoveal region, which covers about 0.1 radians, the numbers would be further divided by a factor of 100, but this would still correspond to perceiving about 1 virtual spider per second. While we do not wish to insist too heavily on a quantitative argument, we want to show that it is not implausible that, even in our everyday experience, the brain may need to encode sensory signals with exceedingly low error probabilities.

One can think of a number of resolutions to this ‘spider-on-the-wall problem’ (changing hotel rooms will not do). Temporal integration, for one, may be used to suppress errors. Also, error rates ought to be influenced by the prior expectation of an event—a quantity we have not included explicitly in our argument. That said, both temporal integration and prior expectation involve trade-offs. Extensive temporal integration requires longer viewing times, and many behaviors need to occur quickly. Relying too heavily upon prior expectation could leave one unable to recognize novel objects.

A more direct way of ensuring reliable discrimination is to employ neural populations that are organized to suppress false alarm (and miss) rates down to extremely low values. In the present paper, we focus on this strategy. As an illustration of the stringency of the requirement, imagine that no more than one virtual spider ought to be perceived in the hour it takes you to fall asleep (as such spider detections could prevent sleep). This condition is satisfied if the false alarm rate remains below 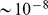 per detection circuit. And of course, the visual system can recognize many objects other than spiders, implying even lower false alarm rates in any one kind of detector so that the total false alarm rate remain very low.

### Other strategies for low-error coding

As was have just explained, infinitesimal error is not a luxury, but a necessity in rapid coding if one wishes to avoid relatively frequent false alarms. We have shown here how correlations can enable population codes to perform with negligible error rates. However, other possible strategies for reducing false alarm errors exist: temporal integration and prior expectation. Both strategies effectively involve raising the detection threshold to suppress the false alarm rate. But both strategies involve trade-offs as well.

First, most stimuli in natural settings are present over periods of time longer than a few tens of milliseconds. Thus, in rapid coding a miss can be corrected: for a miss rate 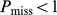 in a fundamental time window of 20 ms, a stimulus present during a period of 200 ms allows 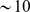 opportunities of detection. These multiple opportunities of detection reduce the overall miss rate to roughly 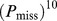, a *much* smaller quantity. However, the consequence is that the false alarm rate, 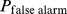 is the short time window, increases to roughly 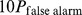 (assuming 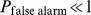) in the long time window. This imbalance can be corrected by raising the detection threshold, 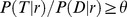 (with 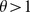 instead of 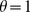), so that the false alarm rate goes down for detection in each fundamental time window. Because the false alarm rate is suppressed exponentially by raising the threshold, but only increased linearly by allowing detection in several successive time bins, such a strategy can be favorable. For instance, in the case of the *independent* code in Fig. 3, if the threshold is raised to boost the miss rate to about 10% (which corresponds to an increase by a factor of 53), then the false alarm rate is reduced from about 0.1% down to 0.0001% (which corresponds to a suppression by a factor of 850). The obvious cost of this strategy is that the presence of new objects in the visual world will be noted slowly, and if there are important objects that require rapid detection this delay and variability in detection may be unfavorable.

Second, prior expectation can modulate the balance between misses and false alarms in a favorable manner. The miss rate and the false alarm rate are weighed by the frequency of occurrence of stimuli, 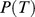 and 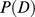 (see Materials and Methods). In practice, these quantities are not known and must be estimated by a freely behaving animal. Changing their values amounts to weighing the two kinds of error—misses and false alarms—by their expectation with regards to the occurrence of stimuli. Mathematically, this is equivalent to weighing miss and false alarm rates as a function of the costs associated with them. Thus, the effects of expectation and cost can both be subsumed in the choice of the decoding boundary, 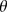. If the boundary is displaced toward the distribution corresponding to Target, then the miss rate increases while the false alarm rate decreases. The reverse occurs if the boundary is displaced toward the distribution corresponding to Distracter. Therefore, an object expected to be incredibly unlikely in a given environment can have its detection threshold raised substantially to prevent unwanted false alarms.

This strategy has the obvious drawback that if the rare object *is* actually present, it will be detected with difficulty. A behaving animal continually updates its internal representations of expectation and cost as a function of experience — a strategy often referred to as Bayesian decision-making. In a new overall visual context, an otherwise rare object may be more likely present, and the animal may consequently lower its detection threshold and, hence, render that object more easily visible. In addition, temporal integration can enhance the detectability of unexpected objects, thus helping to overcome a high detection threshold. But of course, both these methods require more time, so that they will not be effective for rapid detection. Furthermore, there are limits as to how high the miss rate can be allowed to increase without adverse behavioral consequences, which places limits on how effective these strategies can be in achieving very low false alarm rates.

For all these reasons, it is likely that these strategies are combined with population codes having intrinsically low error. In fact, the suppression of the false alarm rate by raising the threshold is much more effective if the distributions of neural activity are already well separated: in the example of the *correlated* code in Fig. 3, increasing the miss rate to 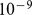 reduces the false alarm rate by another factor of 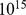.

## Materials and Methods

### Maximum likelihood error bound

In the absence of detailed knowledge about the decoding algorithm employed by readout neurons, we can still establish a bound on performance. This bound is derived from maximum likelihood decoding—an algorithm that minimizes the error rate of deterministic decoding [51]. It assigns Target to a response pattern, 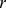, if 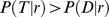 and, conversely, it assigns Distracter to a response pattern, 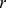, if 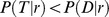, where 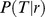 and 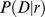 denote the probability that Target and Distracter, respectively, were presented given that the response pattern is 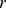. The *miss rate*—the fraction of instances in which Distracter is mistaken for Target—is then calculated as 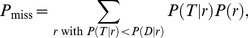
(21)where 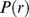 is the probability to record a response pattern 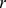 (regardless of the stimulus presented). Similarly, the *false alarm rate*—the fraction of instances in which Target is mistaken for Distracter—is calculated as
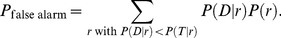
(22)


The *total error rate* committed by maximum likelihood decoding,
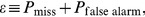
(23)is a lower bound to the error rate committed by any deterministic decoder. Readout neurons make at least 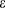 errors per unit time. Throughout, we use the error rate, 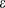, as a measure of the fidelity of the neural population to contrast the coding performance of independent neural populations *versus* correlated neural populations.

Since experiments record the rate of occurrence of neural responses given the stimuli, namely the probabilities 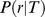 and 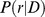, and not the other way around, it is often advantageous to express the miss and false alarm rates in terms of these measurable quantities, as
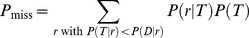
(24)and
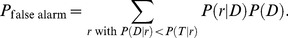
(25)


In the laboratory, 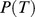 and 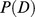 are controlled by the experimenter; in natural situations, 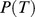 and 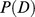 can be thought of as the subject's expectation of the chances of occurrence of the respective stimuli.

In general misses and false alarms are not symmetric, as they represent different kinds of errors. In some situations, one may wish to limit the rate of false alarms more stringently than that of misses, or *vice versa*. A convenient way to impose such a condition is to introduce a threshold, 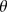, greater or smaller than one, when comparing 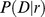 and 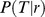, and consequently to generalize the error rates to
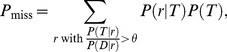
(26)

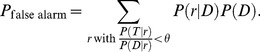
(27)


We discuss the asymmetry between misses and false alarms, and the corresponding role of the threshold, 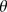, in Discussion.

### Definitions of ‘macroscopic’ and ‘microscopic’ correlations

We consider a neural population divided into 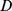 homogeneous pools, labeled by 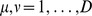, and we call 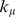 the number of spikes fired in Pool 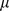 in a given time bin. The ‘macroscopic’ correlation among pools, 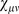, is defined as
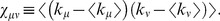
(28)


The ‘microscopic’ variable which characterizes the state of the neural population is 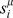; 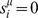 or 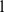
depending upon whether the 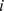
th neuron in Pool 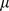 is silent or fires a spike, respectively. The ‘microscopic’ correlation between neuron 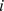 in Pool 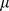 and neuron 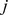 in Pool 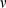 is then defined as
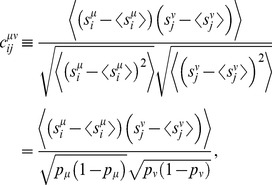
(29)where 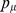 is the firing rate in Pool 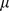.

Since 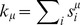, the ‘macroscopic’ correlations are related to the ‘microscopic’ correlations according to
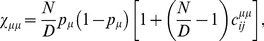
(30)

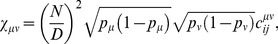
(31)where 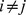, 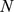 is the total number of neurons in the population and where we have assumed that all pools have the same size. Hence the identity between Eqs. (5) and (8).

### 2-Pool model of correlated neurons: Coding error—numerical treatment

The numerical procedure begins by dividing a population with 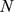 neurons into two homogeneous pools with 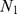 and 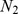 neurons respectively. The maximum entropy distribution over the microscopic variables [47], 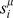, induces a distribution over the spike counts in each pool, 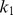 and 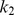, which takes the form
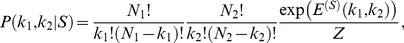
(32)where
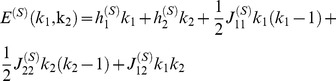
(33)and 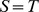 or 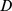 (i.e., 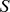 labels the identity of the stimulus). The combinatorial prefactors appear, above, because we consider here the maximum entropy distribution of the microscopic variables (i.e., 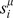, with 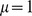
and 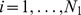 or 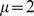
and 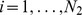, the spiking output of each neuron), rather than the distribution of population variables (i.e., of 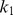 and 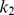, the spiking output in each pool). Thus, the five parameters, 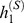, 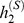, 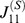, 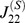, 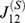, are found by direct numerical solution, such that the firing rates of individual neurons in each other the two pools, 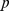 and 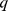, and the normalized pairwise correlations, 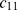 (within Pool 1), 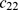 (within Pool 2), 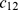 (across Pools 1 and 2), take given values. (Throughout, we borrow symmetric choices (Fig. 2A). That is, in response to Target the firing rates are 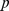 and 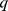 in Pools 1 and 2 respectively, while in response to Distracter the firing rates are swapped, i.e., 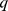 and 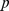, in Pools 1 and 2 respectively. The same holds for the correlation values 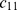 and 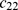.) After finding the maximum entropy distribution corresponding to a given choice of firing rates and pairwise correlations, we used maximum likelihood decoding (described above) to define errors. Specifically, we evaluated Eqs. (24) and (25) for every value of 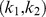 using a threshold of 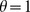 for minimum total error. Thus, our calculation of total error was exact with no approximation made to the decoder's decision boundary.

For the case of a fully heterogeneous population, all of the firing rates and pairwise correlations were randomly perturbed from their homogenous values. For a cell with firing probability 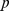, we set its new firing probability to 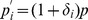, where 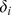 is a Gaussian random variable with vanishing mean and variance 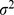. Similarly, for each cell pair with correlation coefficient 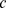, we set its new correlation to 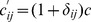, where 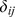 is also a Gaussian random variable with vanishing mean and variance 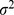. Thus, random instantiations of firing rates and correlations had, on average, the same mean as the matched homogeneous population and a standard deviation of 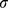, measured as a percentage of the original value. Next, we solved numerically for the pairwise maximum entropy model consistent with the specified firing probabilities and pairwise correlation coefficients. If we denote the activity state of the population by 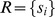, where 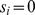 or 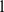 is the activity state of cell 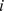, the energy of the full pairwise maximum entropy model reads
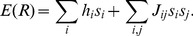
(34)


Because the population size was small (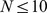), we were able to relate the parameters of the maximum entropy model, 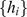 and 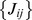, to the firing rates and pairwise correlations, using exact numerical integration over all 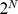 activity states rather than approximating this integral using Monte Carlo methods. Error rates were obtained from maximum likelihood decoding, with the use of the exact decision boundary over all 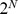 activity states. Clearly, the error rate depended upon the specific random instantiation of firing probabilities and pairwise correlations. In Figs. 4A and B, we show the error rate for 300 random instantiations of heterogeneous populations; in Fig. 4C, we plot the average error rate over all 300 random instantiations along with the standard deviation as an error bar.

The choice of maximum entropy distributions is a reasonable one for establishing upper bounds on the error rate, as these distributions are ‘as spread out as possible’ given the constraints on firing rates and correlations. Strictly speaking, true bounds are obtained from minimum mutual information distributions, but we expect the results to be close to those obtained from maximum entropy distributions. This expectation is substantiated by the results obtained from Gaussian approximations—see the remarks at the end of the next section.

### 2-Pool model of correlated neurons: Coding error—Gaussian approximation

We consider a 2-Pool population with 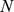 neurons. For the sake of simplicity, we focus on a symmetric case with 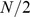 neurons in each pool, firing rates 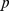 and 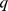 in response to Target and Distracter, respectively, in Pool 1, and vice versa firing rates 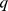 and 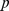 in response to Target and Distracter, respectively, in Pool 2. For the sake of simplicity, also, we specify the calculation to the symmetric case with 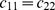, the within-pool correlation coefficients, but the calculation runs along similar lines for more general cases. The pairwise correlation across the two pools is denoted by 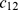. With these hypotheses, a Gaussian approximation to the probability of response to Target reads 
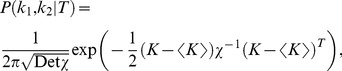
(35)where 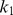 and 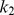 are the spike counts in Pools 1 and 2 respectively. Here, we use the vector notation with
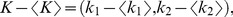
(36)

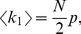
(37)

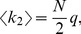
(38)and the covariance matrix
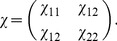
(39)


A similar expression approximates the probability of response to Distracter, but with 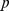 and 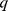 swapped. (The firing rates depend upon the stimulus, but the correlations do not.) For calculational ease, we give a name to the inverse covariance matrix:
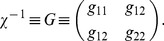
(40)


We calculate the probability of error by integrating the tails of the two distributions, corresponding to the two stimuli, as delineated by the maximum likelihood boundary. For the rather symmetric choice of parameters with which we are concerned here, the maximum likelihood boundary in the 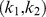-plane is given by the condition
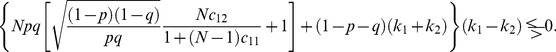
(41)


Thus, the maximum likelihood lies along the diagonal in the 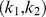-plane; however, the tail of the distribution to be integrated over (in order to obtain the maximum likelihood error) switches from one side of this boundary to the other when the first factor changes sign. What is going on, here, is easy to understand if one considers the angles along which the elongated axes of the distributions are aligned (see Eq. (62), below, for an analytical expression). If the two angles corresponding to the two distributions are not equal, then the two distributions (i.e., their long axes) are not parallel, and ‘they will cross’; at that ‘crossing point’, the maximum likelihood condition switches sign. For several reasons, however, we can safely ignore this complication in the calculation. First, for all cases in which 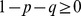, the sign switch occurs for an unphysical negative value of 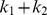; and, indeed, all the examples illustrated in this paper obey the inequality 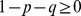 as one would expect for sparse neural responses. Second, we are interested in cases in which the two distributions of neural activity have similar means, and in this case the two elongated distributions in the 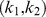-plane are nearly parallel. Indeed, the deviation from a parallel scenario occurs because the firing rates of neurons in response to two stimuli are different (as, otherwise, their correlations do not depend upon the stimulus); this is what yields the stimulus-dependence of the angle of the macroscopic distributions in the 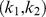-plane. If their means are close, then the distributions are nearly parallel. Finally, in practice, distributions ‘cross’ in any significant way in cases in which they are broad or correlations are unfavorable.

For the remainder of the calculation, it is convenient to parametrize the plane of neural activities in the two pools in coordinates, 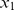 and 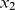, which take the point of maximum equiprobability, 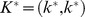, as origin and lie along the maximum likelihood boundary and the orthogonal direction, respectively. Specifically, we set
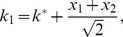
(42)

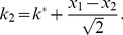
(43)


The error rate is then obtained the 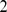-dimensional integral of the probability distribution, with 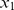 ranging from 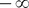 to 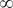 (so that we include a small overestimate that comes from unphysical negative values of the spike counts) and 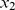 ranging from 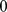 to 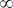. (In order to calculate the total error, we have to take into account both misses and false alarms, i.e., the ‘two tails’ on the two sides of the maximum likelihood boundary. But we also have to normalize this result by the stimulus probability, i.e., by a factor of 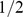.) Thus, 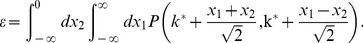
(44)


The probability distribution can be written in terms of the new variables, as
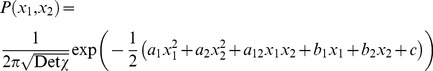
(45)with
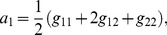
(46)

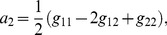
(47)

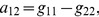
(48)

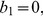
(49)

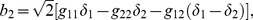
(50)

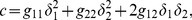
(51)where we have used the shorthand
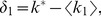
(52)

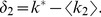
(53)


Performing the Gaussian integral over 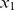, we obtain
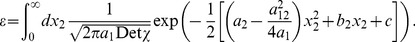
(54)


Finally, this integral can be immediately rewritten as a complementary error function which, in turn, can be expanded:
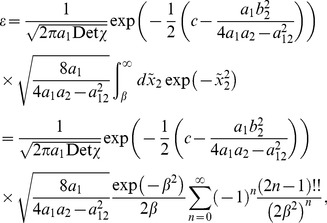
(55)where we have defined
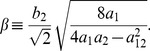
(56)


Finally, keeping only the dominant term and simplifying the expression, we compute the error according to
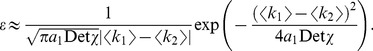
(57)


We obtain Eq. (1), (2), and (3) when we replace 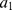 and Det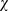 by their expressions in terms of firing rates and correlation coefficients.

We note that, above, we have simply integrated the tails of the distributions as delineated by the maximum likelihood bound. In simple (symmetric) cases, this can be recast as a linear estimation problem, and the error rate can be related to the 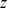
- (or 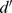-) score. Furthermore, the term appearing in the argument of the exponential is closely related to the linear Fisher information, and can be intuited as such.

We emphasize the agreement between the numerical and the analytical results (dots versus solid lines in Figs. 2A-D and Figs. 3A and C), which is not to be expected in general and is encouraging here. Indeed, numerical results are derived by making use of maximum entropy distributions. These are as broad as the constraints on firing rates of individual neurons and pairwise correlations allow, yet when expressed in terms of spike counts their tails fall off more rapidly than Gaussian tails. Estimations of the error rate from maximum entropy distributions and from Gaussian distributions coincide. We recall that the maximum likelihood error is dominated by the height of the distributions at equiprobability. So the quantitative similarity between numerical and Gaussian results means that, even for very stringent error thresholds, the asymptotic behavior of the tails does not play a dominant role.

### Robustness of high-fidelity coding with respect to parameter variations

**Figure 9:**
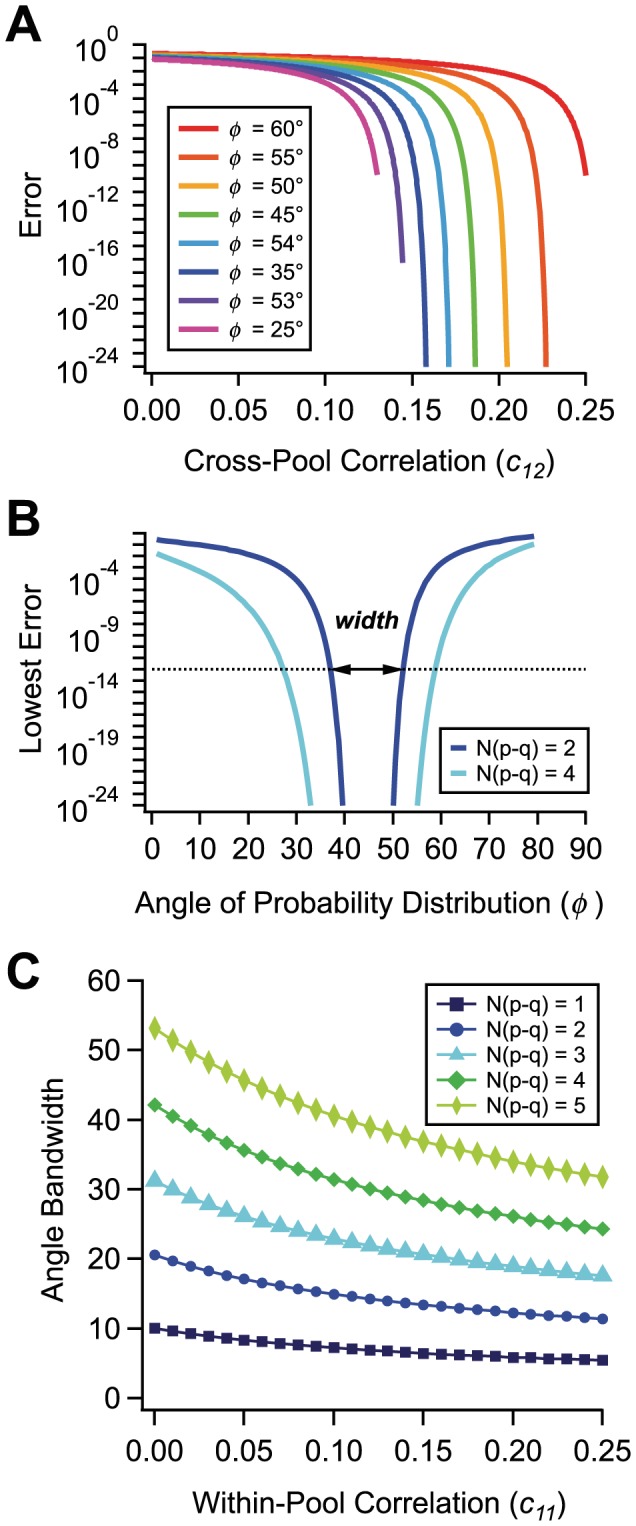
Robustness to parameter variations. **A.** Probability of error as a function of the cross-pool correlation 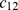 for populations with 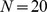 neurons and different angles 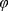 of their probability distributions in the space of 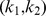; parameters are (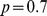, 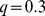, 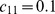) with 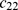 set to give the chosen angle (Eq. (62)). **B.** Probability of error as a function of angle for fixed difference in spike count, 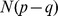, intersects the error criterion 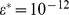 at two angles, which defines the angular bandwidth. **C.** Angular bandwidth plotted as a function of within pool correlation, 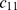, for different values of the difference in spike count, 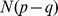.

High-fidelity coding results from the suppression of overlap among response probability distributions corresponding to different stimuli. By tuning one combination of the correlation parameters, distributions become thin (*i.e.*, favorable), and we have demonstrated that this can occur for realistic values of the correlations. But even in the singular limit of infinitely thin (*i.e.*, locked-in) distributions, independent parameters are left free, namely, the orientations of the principal axes of the distributions or, equivalently, the angles along which the elongated distributions lie in the 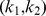 plane. We have denoted this angle by 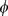 (Fig. 1B). An important question is whether these parameters have to be fine-tuned for high-fidelity coding. We show, here, that no fine-tuning is necessary: high-fidelity coding operates over a wide range of parameter choices (Fig. 9).

Consider, for example, the dependence of the error rate upon the cross-pool correlation strength, 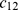, for several choices of the angle 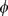 (Fig. 9A). Clearly, when the two distributions corresponding to Target and Distracter are elongated along the same direction (here the diagonal, 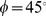, because of our choice of symmetric parameters), the error rate plunges down to vanishing numbers for appropriate correlation values. If the two distributions are not parallel, there always remains some overlap, even if they are infinitely thin. However, this overlap is so small that, even when the angle differs from the diagonal by as much as 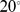, the error rate is suppressed by more than ten orders of magnitude (Fig. 9A).

In order to explore the parameter dependence of the error rate, we set a (small) ‘error rate threshold’, 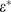, not to be exceeded. The closer 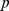 and 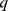 are, *i.e.*, the more similar the mean responses to Target and the response to Distracter, then the more stringent becomes the threshold condition, 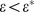, upon the parameters of the model. An arbitrary threshold—here, we choose 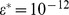
—defines a corresponding ‘angle bandwidth’: a range of distribution angles, 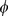, within which the error rate remains below threshold (Fig. 9B). We selected the value of the error threshold to be sufficiently low that networks within the angle bandwidth contribute fewer than a single error per human lifetime. Clearly, the angle bandwidth depends upon all other model parameters. The closer the firing rates 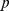 and 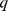 in response to Target and Distracter respectively, the closer the two distributions lie and, hence, the more precisely their angle has to be tuned for error rate suppression. Yet, even when the average activities in the two pools differ by as little as two to five spikes, the angle bandwidth remains as large as 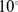 to 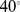 over a wide range of correlation values (Figs. 9B and C). Thus, error rate suppression is robust to small parameter variations.

### Arguments for lock-in beyond a Gaussian approximation

Here, we present general arguments on the role of correlation in high-fidelity coding, which do not rely on a Gaussian approximation of probability distributions. We assume only that the probability distributions of spike counts in response to Target and Distracter are ‘well-behaved’; specifically, that they each have a single maximum and that their tails decay rapidly enough. Then the knowledge of the correlation structure is sufficient to discuss the degree of their overlap and, hence, the coding error rate. For the sake of simplicity we still consider a 2-Pool model, but our arguments can be transposed to the general case of a 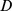-Pool model.

We start by examining the quantity
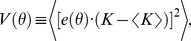
(58)where 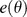 is a unit vector along the direction given by the angle 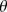 and 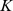 is the vector of spike counts. This quantity is calculated as
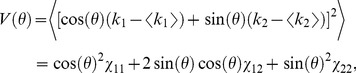
(59)
*i.e.*, it is the variance along the direction prescribed by the unit vector 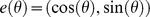 in the 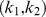-plane of spike counts. Optimizing 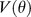 with respect to the rotation angle, we find that it reaches its minimal and maximal values,
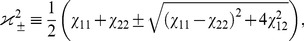
(60)along the two orthogonal angles given by
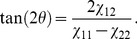
(61)


This expression can also be written in terms of the microscopic correlations as
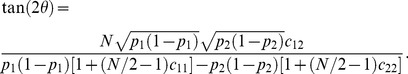
(62)


For positive correlation, the angle along which the distribution elongates, 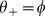 (Fig. 1B), lies between 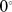 and 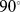. The other solution of this equation lies at right angle with 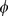, 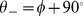, and defines the direction of ‘probability compression’. The quantities that govern overlap suppression are the small variances, 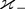, and the angles 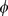, for each of the two distributions corresponding to Target and Distracter. The error rates decrease with smaller 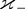 and more parallel distributions.

The positivity of 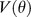 implies a constraint upon the values of the macroscopic correlations: 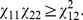
(63)


In terms of the microscopic correlations, the inequality reads
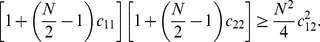
(64)


This condition amounts to the positivity of probability. When equality is achieved, the corresponding probability distribution becomes infinitely thin along one direction, *i.e.*, the probability of any state in the 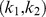-plane away from this line vanishes. When equality is achieved, we say that the neural population is ‘*locked-in*’; in this case, the coding error rate can vanish. When correlation values are such that the inequality is satisfied, and hence the coding error rate can be massively suppressed, we refer to the pattern of correlation as ‘*favorable*’.

We note in passing that a vanishing error in the Gaussian approximation, *i.e.*, 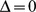 (see Eq. (2)), corresponds to two ‘infinitely thin’ probability distributions whose directions of largest variance are parallel. Indeed, the condition 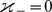, which occurs when 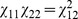, together with the condition 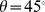 (see Eq. (61) above) imply
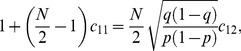
(65)

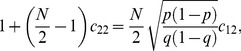
(66)
*i.e.*, 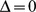.

### 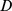-Pool model of independent neurons: Coding capacity

For an estimate of the coding capacity of a population of independent neurons, we approximate the spike count distribution by a Gaussian with appropriate mean and variance. In the 1-Pool case with 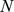 neurons, this distribution reads
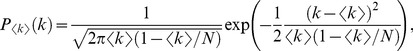
(67)where 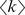 is the mean spike count and 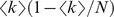 the variance. We then ask, given one such distribution with parameter 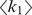, how far away along the 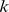-line should a distribution, with parameter 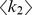, be placed so that the probability not exceed a small value, 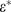, a the point of equiprobability, 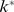: 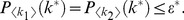
(68)


If this bound is achieved, the form of Eq. (67) implies the relation
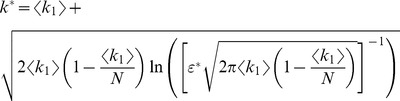
(69)for 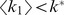. Since 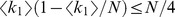 and 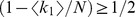 if 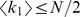, we obtain
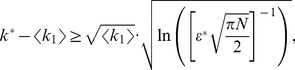
(70)i.e., a lower bound on the distance between the mean of the distribution and the point of equiprobability. Similarly, for 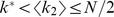, we have
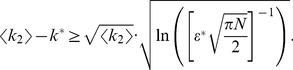
(71)


Combining the two inequalities, we obtain a lower bound on the distance between the means of the two distributions, as
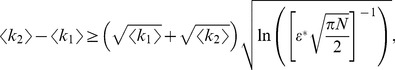
(72)or
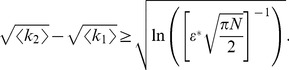
(73)


We can then iterate this argument for successive distributions, corresponding to different stimuli, and for each pair of distributions the bound
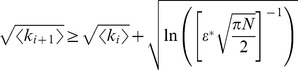
(74)holds, up to 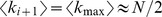. For a total of 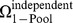 distributions to my fit along the 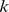-axis, the means of half of these will be between 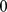 and 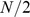, while the other half will be between 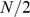 and 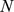. Thus,
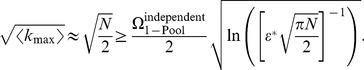
(75)


From this relation, we obtain the final bound on the capacity of a homogeneous population of independent neurons, as
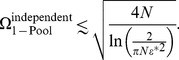
(76)


In the 2-Pool case, we calculate similarly the number, 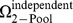, of well-separated probability distributions that can be fit within the positive quadrant of the 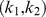-plane of spike counts. Here, 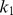 and 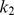 each run from 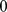 to 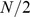, so 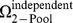 is roughly evaluated as 
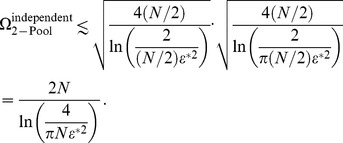
(77)


Similarly, in the general 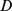-Pool case, each axis of the response space runs from 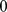 to 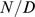, so that
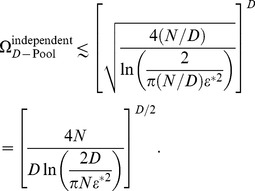
(78)


By analogy with a population of 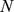 deterministic neurons, we define the capacity per neuron, 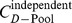, as
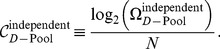
(79)


In the deterministic case, the population as a whole codes for 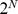 states and the capacity per neuron is equal to 1 bit. In the case of independent, but stochastic, neurons,
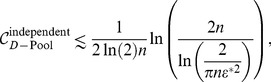
(80)where
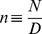
(81)is the number of neurons per pool. The capacity decreases with decreasing 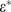. For a given value of 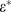, the capacity is maximal for a characteristic pool size which depends upon 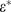 but does not depend upon 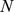 and which can be calculated perturbatively. Indeed, the minimization of the capacity yields the optimal pool size as the implicit solution of the equation
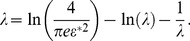
(82)


Solving this equation perturbatively to the next-to-lowest order, we obtain an approximate optimal pool size, as
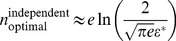
(83)and a maximal capacity per neuron given by
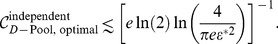
(84)


Equivalently, the number of stimuli that a population of 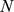 independent neurons can encode with an error threshold 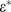 is limited by
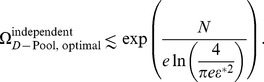
(85)


### 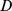-Pool model of correlated neurons: Coding capacity

We derive an estimate of the capacity in the correlated case by evaluating how many ‘thin probability distributions’ can be fitted in the quadrant of possible response patterns defined by 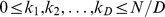. In a 2-Pool population (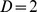), we can arrange one row of ‘parallel distributions’ along the diagonal that connects the points 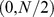 and 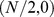 in the 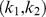 plane. (Three such rows are displayed in Fig. 6B.) If neighboring distribution centers differ by 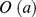 spike, this manipulation yields a number
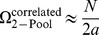
(86)of well separated probability distributions that the population can code for. Similarly, in the general 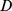-Pool case we arrange a set of correlated distributions across a hyperplane within the hypercube with edge 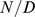 in the 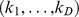 space. Such a configuration immediately yields a scaling
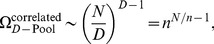
(87)where
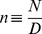
(88)is the number of neurons per pool, as before. To be more precise, we can bound 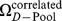 from below. If we are concerned that distributions may overlap near the faces of the hypercube, we can, for example, allow them to fill only a central half of the hyperplane. Furthermore, if neighboring distribution centers are separated by 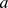 spikes, we obtain
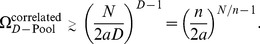
(89)


This quantity behaves differently from its counterpart in the independent case: for a wide range of even vanishingly small error thresholds, 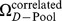 is essentially independent of the error threshold as realistic values of the correlation coefficients can be chosen so as to make the distributions much narrower than 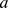. For fixed 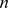, this bound scales with 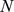 in a trivial manner akin to the independent case. Indeed, the capacity per neurons,
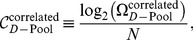
(90)here becomes
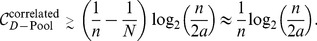
(91)


The capacity per neuron is maximized for
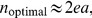
(92)where 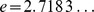 is Euler's number, and is evaluated as
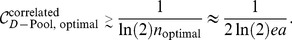
(93)


We find
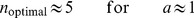
(94)and
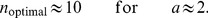
(95)


Correspondingly,
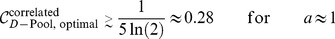
(96)and
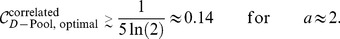
(97)


As opposed to the case of independent neurons, here one does not need to invoke large values of 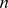 for low-error coding. This is because 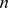 is not the only parameter from which the system can take advantage to suppress error rates; for each value of 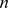, the correlation coefficients may be tuned to suppress error rates. We emphasize that the result for optimality, with 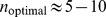, is self-consistent: low-error coding can indeed occur with such small pool sizes (see Fig. 3).

We find that, in a correlated population, each neuron can carry as much as 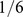 to 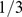 bits of information. This result is to be contrasted with the absolute maximum of 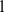 bit of information in the case of independent, deterministic neurons and with the corresponding result for independent, stochastic neurons, Eq. (84). In the correlated case, the optimal capacity per neuron is fixed, whereas in the independent case it drops with 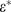. In particular, from Eqs. (84) and (93) with 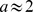, we conclude that individual neurons are more informative in a correlated population, as compared to an independent population, as soon as the error rate threshold, 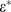, falls below 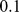. Thus, for any realistically small value of the error rate threshold, correlated populations are favored.

Taking the 2-Pool model as an example, we note that only for relatively large values of the parameters (e.g., 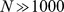 or 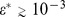) does 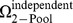 compare with 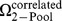. At relatively low threshold values (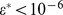), 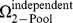 remains well below 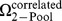 for any reasonable (and even large) value of the population size (Fig. 5D), as the behavior of 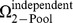 is dominated by 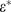 rather than by 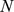 (Fig. 5D). This behavior obtains because the nearly isotropic tails of the distributions for independent neurons forbid the presence of more than one or a few distribution centers within the space of neural responses, if the error threshold is stringent.

It is worth mentioning that for loose error thresholds 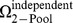 may exceed 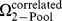. This results from the fact that independent distributions are arranged on a two-dimensional grid, whereas correlated distributions, which are compressed along one direction, are arranged along a line (along the ‘compressed direction’). Thus, independent distributions can take advantage of the 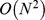 possible positions of their centers, whereas correlated distributions have only 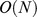 choices.

## Supporting Information

Text S1 **High-Fidelity Coding with Correlated Neurons–supplementary material.** Supplementary methods. Supplementary discussion, with one supplementary figure.
(PDF)
