# Supplementary material for: High-Fidelity Coding with Correlated Neurons
Source: PLoS Comput Biol. 2014 Nov 20;10(11):e1003970. doi: 10.1371/journal.pcbi.1003970 (PMC4238954; doi:10.1371/journal.pcbi.1003970)
Supplement: Text S1 — High-Fidelity Coding with Correlated Neurons–supplementary material. Supplementary methods. Supplementary discussion, with one supplementary figure. (PDF) [file pcbi.1003970.s001.pdf]

High-Fidelity Coding with Correlated Neurons
Supplementary Discussion

Rava Azeredo da Silveira
Department of Physics,
Ecole Normale Supérieure, 24 rue Lhomond, 75005 Paris, France
Laboratoire de Physique Statistique,
Centre National de la Recherche Scientifique, Université Pierre et Marie Curie, Université Denis Diderot, France
Princeton Neuroscience Institute,
Princeton University, Princeton, New Jersey 08544, U. S. A.

Michael J. Berry II
Department of Molecular Biology and Princeton Neuroscience Institute,
Princeton University, Princeton, New Jersey 08544, U. S. A.

1 Arguments for the detrimental effect of positive correlation on coding with a homogeneous neural population

It is often said that positive correlation is detrimental to coding. This claim is based on intuition developed for homogeneous populations [13, 16], as we explain in this section. Imagine turning on positive correlation in the population response to Target (Supplementary Fig. 1A). Distributions of population activity for increasing values of correlation are progressively wider, causing greater overlap and hence an enhanced coding error rate. This behavior is generic for positive values of the correlation (Supplementary Fig. 1B). (In extreme, non-generic cases with very large values of the correlation, the distribution corresponding to Target may become bimodal and concentrated around 0 and N. The overlap between the two distributions can then decrease, and hence coding can improve. But such extreme cases are very different qualitatively from the experimental situation in which pairwise correlations are small to moderate, ranging from -0.1 to 0.5 [3, 4, 8, 9, 10]. In contrast to positive values of the correlation, negative values reduce the discrimination error but, again, such values are rarely observed experimentally.)

Simple arguments explain this behavior. Positive correlations enhance fluctuations in the population response, as compared to the independent case, and, as a result, suppress the signal-to-noise ratio. If r_i denotes the response of

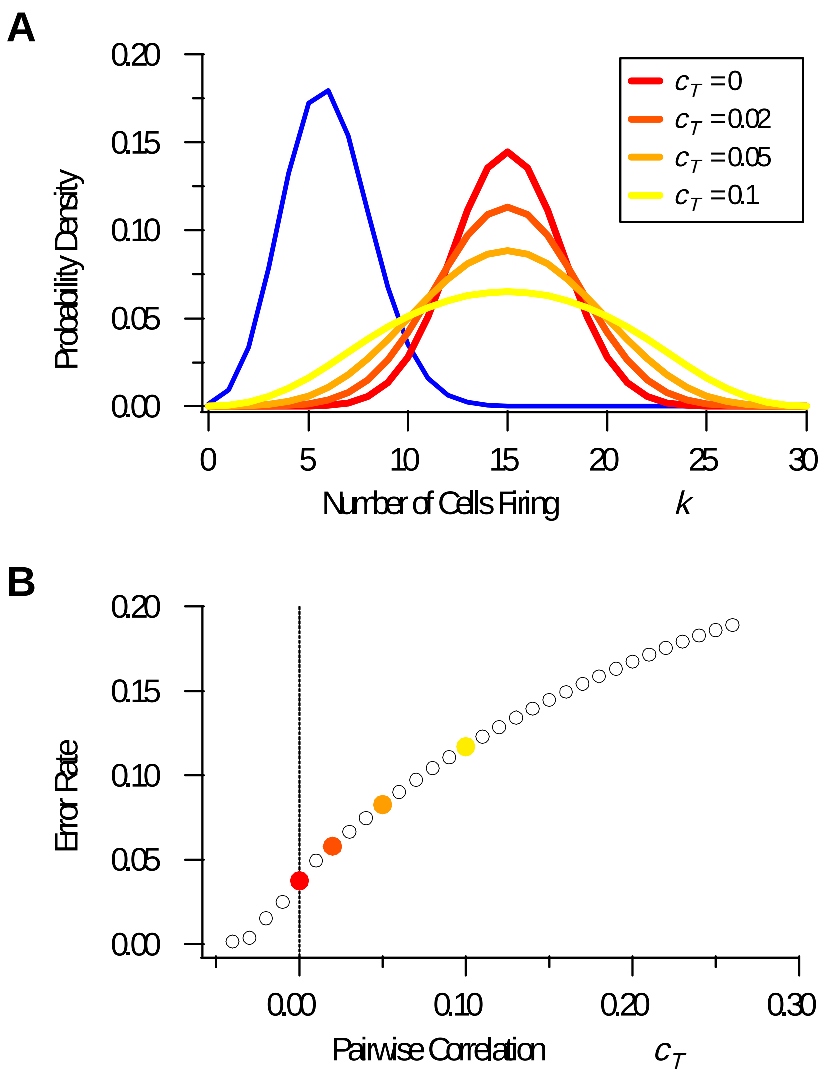

Figure 1: **Homogeneous Populations.** **A.** Probability distribution of the spike counts k in a homogeneous population given the Distracter stimulus (blue) and the Target stimulus with different values of pairwise correlation, c_T (shown by color); parameters are $N = 30$ neurons, $p_T = 0.5$, $p_D = 0.2$. **B.** Probability of error as a function of the pairwise correlation during the Target stimulus, c_T ($N = 30$ neurons, $p_T = 0.5$, $p_D = 0.2$, $c_D = 0$), with examples from panel A (dots with color matching panel A).

neuron i, the variance of the population activity is

$$\left\langle \left(\sum_i r_i - \left\langle \sum_i r_i \right\rangle \right)^2 \right\rangle = \sum_i \left\langle (r_i - \langle r_i \rangle)^2 \right\rangle + \sum_{i \neq j} \langle (r_i - \langle r_i \rangle)(r_j - \langle r_j \rangle) \rangle , \tag{1}$$

where brackets indicate an average over trials. The first sum on the right-hand-side pertains to fluctuations in single-neuron responses and is non-vanishing in both independent and correlated cases. The second sum on the right-hand-side pertains to correlations among neurons. For negative correlations (anti-correlations), this sum is negative and, hence, the distribution of neural activity is more narrowly peaked than in the independent case. By contrast, positive correlations broaden the distribution. In the anti-correlated case, distributions of population activity corresponding to different stimuli tend to be well separated, while in the positively correlated case, overlaps tend to be greater. Therefore, homogeneous populations with positive correlation have worse coding performance than corresponding independent populations and, consequently, require more neurons to achieve a low rate of coding errors.

We can understand the hindrance of coding performance from positive correlations in an alternate, simple fashion. A homogeneous population with positive correlation behaves, effectively, as a smaller population. In the limiting case of a perfectly correlated population in which all neurons respond identically, the entire population behaves as one, big neuron. Hence, we expect such positively correlated populations to code information with less 'resolution' and, consequently, to commit coding errors more often than corresponding independent populations do.

2 High-fidelity coding bare bones

In the companion paper we demonstrate, quantitatively and with the use of simple models, that positive correlation can suppress coding errors and enhance coding capacity massively. The basic mechanism behind this effect was noted by a number of authors [7, 15, 1, 13, 2] and is simple to understand: positive correlations can deform the shape of probability distributions of neural activity in such a way as to sharpen the distinction between nearby probability distributions (Fig. 1B). Put differently, while positive correlations have a broadening effect overall, they can nonetheless suppress the tails of probability distributions along relevant directions, thereby reducing the unfavorable effect of neural variability.

The same idea can be expressed in a more general fashion: the structure of correlation can be such that it relegates noise into a non-informative mode of the neural population response. A simple example provides a nice illustration ([11]; a similar argument is presented in Ref. [1]). Consider two neurons with

responses

$$r_1 = m_1 + \delta_1, \quad (2)$$
$$r_2 = m_2 + \delta_2. \quad (3)$$

We assume that the mean responses, m_1 and m_2, are different, such that m_1 (m_2) is large (small) in response to the Target stimulus, and *vice versa* for the Distracter stimulus. The additive variabilities, δ_1 and δ_2, are highly correlated, such that $\delta_1 \approx \delta_2$. Then the informative mode,

$$r_- \equiv r_1 - r_2 \approx m_1 - m_2, \quad (4)$$

is close to noiseless, while all the noise is relegated to the uninformative mode,

$$r_+ \equiv r_1 + r_2 \approx m_1 + m_2 + \delta_1 + \delta_2. \quad (5)$$

Our results can also be viewed in terms of a similar mechanism: informative and uninformative modes correspond to combinations of pool spike counts, the k_is, and given patterns of positive correlations relegate variability to the uninformative modes. In the simplest, symmetric, 2-pool model, correlation sharpens the response distributions along the informative mode, $k_1 - k_2$, while it blurs them along the uninformative mode, $k_1 + k_2$. Clearly, it is a signature of correlated coding that informative modes can be identified only when simultaneous activities of the neurons in the population are considered.

3 Learning in read-out circuits

The fact that realistic values of correlation can yield highly accurate coding in small population suggests a picture in which individual neurons in a 'higher-level' area read out information from a very small subset of the neurons in the 'lower-level' area. The question then becomes: how does the brain find the appropriate neurons form which to extract relevant information? This question is unsolved, but experiments on brain-machine interfaces demonstrate that the brain has a truly remarkable ability to change its circuitry in sensory-motor pathways to activate the relevant motor neurons. In an experiment, ~ 100 neurons in primary motor cortex were recorded and their responses were used to drive movements of cursors or even robotic arms with simple cosine tuning functions [14]. Under these circumstances, monkeys were able to achieve high performance in directing movements. Most impressively, the authors showed that they could re-arrange the tuning curves used to translate neural activity into movements of the robotic arm, and monkeys could change their entire sensory-motor pathway in order to fire those particular neurons in the right pattern to achieve the desired movement [6]. In many cases, the tuning curves were completely inverted, and yet the monkeys re-learned how to fire those neurons appropriately. Analogous results have been reproduced by another lab [5]. A related example comes from experiments in which human subject wear

inverting prism glasses. Initially, the world appears upside-down, resulting in profound motor deficits and disorientation. But after about a week, subjects regain their coordination, evidently requiring a complete remapping (inversion) of visual stimuli to motor outputs [12].

So, while the brain faces great difficulties in obtaining useful information encoded by sensory circuits and must be subject to certain limits in accomplishing these, it is clear that the brain has a remarkable ability to surmount these difficulties in many situations. We currently have very little understanding of how the brain manages this, and hence we really don't know at this point what the limitations are.

References

[1] L. F. Abbott and P. Dayan. The effect of correlated variability on the accuracy of a population code. *Neural Comput*, 11(1):91–101, 1999.

[2] B. B. Averbeck, P. E. Latham, and A. Pouget. Neural correlations, population coding and computation. *Nat Rev Neurosci*, 7(5):358–66, 2006.

[3] W. Bair, E. Zohary, and W. T. Newsome. Correlated firing in macaque visual area mt: time scales and relationship to behavior. *J Neurosci*, 21(5):1676–97, 2001.

[4] J. Fiser, C. Chiu, and M. Weliky. Small modulation of ongoing cortical dynamics by sensory input during natural vision. *Nature*, 431(7008):573–8, 2004.

[5] Karunesh Ganguly and Jose M Carmena. Emergence of a stable cortical map for neuroprosthetic control. *PLoS biology*, 7(7):e1000153, 2009.

[6] Beata Jarosiewicz, Steven M Chase, George W Fraser, Meel Velliste, Robert E Kass, and Andrew B Schwartz. Functional network reorganization during learning in a brain-computer interface paradigm. *Proceedings of the National Academy of Sciences*, 105(49):19486–19491, 2008.

[7] K. O. Johnson. Sensory discrimination: decision process. *J Neurophysiol*, 43(6):1771–92, 1980.

[8] A. Kohn and M. A. Smith. Stimulus dependence of neuronal correlation in primary visual cortex of the macaque. *J Neurosci*, 25(14):3661–73, 2005.

[9] E. J. Lang, I. Sugihara, J. P. Welsh, and R. Llinas. Patterns of spontaneous purkinje cell complex spike activity in the awake rat. *J Neurosci*, 19(7):2728–39, 1999.

[10] D. Lee, N. L. Port, W. Kruse, and A. P. Georgopoulos. Variability and correlated noise in the discharge of neurons in motor and parietal areas of the primate cortex. *J Neurosci*, 18(3):1161–70, 1998.

[11] J.-P. Nadal. Private communication.

[12] Hans Richter, S Magnusson, K Imamura, M Fredrikson, M Okura, Y Watanabe, and B Långström. Long-term adaptation to prism-induced inversion of the retinal images. *Experimental brain research*, 144(4):445–457, 2002.

[13] H. Sompolinsky, H. Yoon, K. Kang, and M. Shamir. Population coding in neuronal systems with correlated noise. *Phys Rev E Stat Nonlin Soft Matter Phys*, 64(5 Pt 1):051904, 2001.

[14] Meel Velliste, Sagi Perel, M Chance Spalding, Andrew S Whitford, and Andrew B Schwartz. Cortical control of a prosthetic arm for self-feeding. *Nature*, 453(7198):1098–1101, 2008.

[15] R. Vogels. Population coding of stimulus orientation by striate cortical cells. *Biological Cybernetics*, 64:25–31, 1990.

[16] E. Zohary, M. N. Shadlen, and W. T. Newsome. Correlated neuronal discharge rate and its implications for psychophysical performance. *Nature*, 370(6485):140–3, 1994.
